# Epidemiology of antimicrobial resistance (AMR) on California dairies: descriptive and cluster analyses of AMR phenotype of fecal commensal bacteria isolated from adult cows

**DOI:** 10.7717/peerj.11108

**Published:** 2021-04-20

**Authors:** Essam M. Abdelfattah, Pius S. Ekong, Emmanuel Okello, Tapakorn Chamchoy, Betsy M. Karle, Randi A. Black, David Sheedy, Wagdy R. ElAshmawy, Deniece R. Williams, Daniela Califano, Luis Fernando Durán Tovar, Jonathan Ongom, Terry W. Lehenbauer, Barbara A. Byrne, Sharif S. Aly

**Affiliations:** 1 Veterinary Medicine Teaching and Research Center, School of Veterinary Medicine, University of California, Davis, Tulare, CA, USA; 2 Department of Animal Hygiene, and Veterinary Management, Faculty of Veterinary Medicine, Benha University, Moshtohor, Qalyubia, Egypt; 3 Department of Epidemiology, National Veterinary Research Institute, Vom, Plateau State, Nigeria; 4 Department of Population Health & Reproduction, School of Veterinary Medicine, University of California, Davis, Davis, CA, USA; 5 Cooperative Extension, Division of Agriculture and Natural Resources, University of California, Davis, Orland, CA, USA; 6 Cooperative Extension, Division of Agriculture and Natural Resources, University of California, Davis, Santa Rosa, CA, USA; 7 Department of Internal Medicine and Infectious Diseases, Cairo University, Giza, Giza, Egypt; 8 Department of Pathology, Microbiology, and Immunology, School of Veterinary Medicine, University of California, Davis, Davis, CA, USA

**Keywords:** Antimicrobial resistance, Dairy cattle, *Escherichia coli*, *Enterococcus* spp, Multidrug resistance, Phenotype, California, Region, Season, Cohort study

## Abstract

**Background:**

This study describes the occurrence of antimicrobial resistance (AMR) in commensal *Escherichia coli* and *Enterococcus/Streptococcus* spp. (ES) isolated from fecal samples of dairy cows and assesses the variation of AMR profiles across regions and seasons following the implementation of the Food and Agricultural Code (FAC) Sections 14400–14408 (formerly known as Senate Bill, SB 27) in California (CA).

**Methods:**

The study was conducted on ten dairies distributed across CA’s three milk sheds: Northern California (NCA), Northern San Joaquin Valley (NSJV), and the Greater Southern California (GSCA). On each study dairy, individual fecal samples were collected from two cohorts of lactating dairy cows during the fall/winter 2018 and spring/summer 2019 seasons. Each cohort comprised of 12 cows per dairy. The fecal samples were collected at enrollment before calving (close-up stage) and then monthly thereafter for four consecutive time points up to 120 days in milk. A total of 2,171 *E. coli* and 2,158 ES isolates were tested for antimicrobial susceptibility using the broth microdilution method against a select panel of antimicrobials.

**Results:**

The *E. coli* isolates showed high resistance to florfenicol (83.31% ± 0.80) and sulphadimethoxine (32.45%), while resistance to ampicillin (1.10% ± 0.21), ceftiofur (1.93% ± 0.29), danofloxacin (4.01% ± 0.42), enrofloxacin (3.31% ± 0.38), gentamicin (0.32% ± 0.12) and neomycin (1.61% ± 0.27) had low resistance proportions. The ES isolates were highly resistant to tildipirosin (50.18% ± 1.10), tilmicosin (48% ± 1.10), tiamulin (42%) and florfenicol (46% ± 1.10), but were minimally resistant to ampicillin (0.23%) and penicillin (0.20%). Multidrug resistance (MDR) (resistance to at least 1 drug in ≥3 antimicrobial classes) was observed in 14.14% of *E. coli* isolates and 39% of ES isolates. *Escherichia coli* isolates recovered during winter showed higher MDR prevalence compared to summer isolates (20.33% vs. 8.04%). A higher prevalence of MDR was observed in NSJV (17.29%) and GSCA (15.34%) compared with NCA (10.10%).

**Conclusions:**

Our findings showed high rates of AMR to several drugs that are not labeled for use in lactating dairy cattle 20 months of age or older. Conversely, very low resistance was observed for drugs labeled for use in adult dairy cows, such as cephalosporins and penicillin. Overall, our findings identified important differences in AMR by antimicrobial class, region and season.

## Introduction

Disease prevention and control using antimicrobial drugs (AMD) continue to play a unique and vital role in maintaining dairy cattle health. However, with the use of AMD comes the risk of antimicrobial resistance (AMR) affecting both dairy cattle and human populations (McManus & Pharm, 1997). As a result, AMR has become a substantial global public health concern that mandates collaborative work between public health and veterinary medicine. To control and reduce AMR, the US Food and Drug Administration (FDA) published its guidelines regulating therapeutic use in feed and water of Medically Important Antimicrobial Drugs (MIADs) in food-producing animals and prohibited these antimicrobials for production purposes, such as growth promotion and feed efficiency. The Veterinary Feed Directive (VFD) final rule issued by FDA was implemented in January 2017 and mandated supervision by a licensed veterinarian for use of MIADs in feed or water under a valid Veterinarian Client Patient Relationship (VCPR) (U.S Food and Drug Administration (FDA), 2015). Soon after, on 1 January 2018, California (CA) implemented the Food and Agricultural Code (FAC) Sections 14400–14408 (FAC (Food and Agricultural Code), 2015), formerly known and here onwards referred to as Senate Bill 27 (SB 27), making it the first state in the US to remove all MIADs from over-the-counter use to requiring veterinary oversight and prescription.

California is the leading dairy producing state in the United States, with over 1.7 million dairy cows producing 18.5 percent of the nation’s milk supply (18.1 billion kilograms of milk) on 1,331 dairies (California Department of Food and Agriculture (CDFA), 2018). The current study was conducted after implementation of SB 27 to provide baseline data for future evaluation of the impact of new regulations on AMR and AMD use on CA dairies. The SB 27 law increased veterinary oversight for the use of all other dosage forms of MIADs used in livestock, development of voluntary AMD stewardship guidelines, and best management practices for veterinarians, as well as livestock owners and their employees who are involved with administering medically important antimicrobial drugs. In addition, SB 27 mandates monitoring of sales and usage of AMD, resistant bacteria and livestock management practices.

Understanding the patterns of AMR in CA dairies across regions and seasons will further our understanding of potential challenges encountered by the dairy industry as they implement AMD stewardship practices. Fecal commensal bacteria such as *E. coli* have been widely used as indicator organisms for monitoring AMR for a wide range of bacterial species including pathogens (Barlow et al., 2017; Benedict et al., 2015; Carson et al., 2008). *Escherichia coli* is a Gram-negative commensal bacterium that is a good indicator for AMR (Schroeder et al., 2002) because it acquires resistance rapidly (Von Baum & Marre, 2005). Gram-positive bacteria including *Enterococcus* spp. and *Streptococcus* spp. are commonly found as normal inhabitants of the gastrointestinal tracts of animals and humans. Occasionally, *Enterococcus* spp. cause opportunistic infections in humans and animals, including urinary tract and wound infections, bacteremia and endocarditis (Arias & Murry, 2008). Therefore, monitoring the AMR of *Enterococcus* spp. and *Streptococcus* spp. may provide insight into AMR of bacterial species from dairy cows. Fostering a better understanding of the complex pathways between on-farm drug use and AMR in dairy cattle populations is vital to mitigating AMR through antibiotic stewardship and judicious use.

Our hypothesis was that AMR phenotypes of commensal bacteria isolated from fecal samples collected from adult dairy cows on CA dairies vary by herd demographics, management practices and AMD practices. A second hypothesis was that fecal commensal bacteria are more resistant to AMD commonly used in adult cows compared to drugs commonly used in youngstock. Our objective was to describe the prevalence and patterns of AMR phenotypes among commensals isolated from fecal samples of adult dairy cows followed from close-up to 120 days in milk on 10 CA dairies across varied regions and seasons. Results of this study will provide clinicians and veterinarians with epidemiological insights on the prevalence of bacterial resistance against commonly used AMDs which will inform their choices for selection of AMD for treatment of diseases, and guide stewardship practices that reduce spread of AMR. Finally, AMR estimates reported here serve as a baseline for future monitoring of antibacterial resistance in bacteria from adult dairy cows.

## Materials and Methods

### Study herds

The study was approved by the University of California Davis’s Institutional Animal Care and Use Committee (protocol number 19871). As part of a larger surveillance project conducted in CA, dairies were identified both through a voluntary AMD use stewardship survey mailed to CA’s registered Grade A milk producing dairies, in which producers also indicated an interest in this on-farm study, and through the investigator’s contact networks with the dairy industry (extension, dairy producers, and veterinary practitioners). Herds were eligible for enrollment if their management kept records of all AMD treatments and were willing to voluntarily participate in the year-long study. Enrolled dairies had to also allow study personnel access to their adult cows for fecal sampling, dairy cattle treatment and production records, and all dispensed AMD vials during the year-long study period which will be the subject of forthcoming publications.

**Figure 1:**
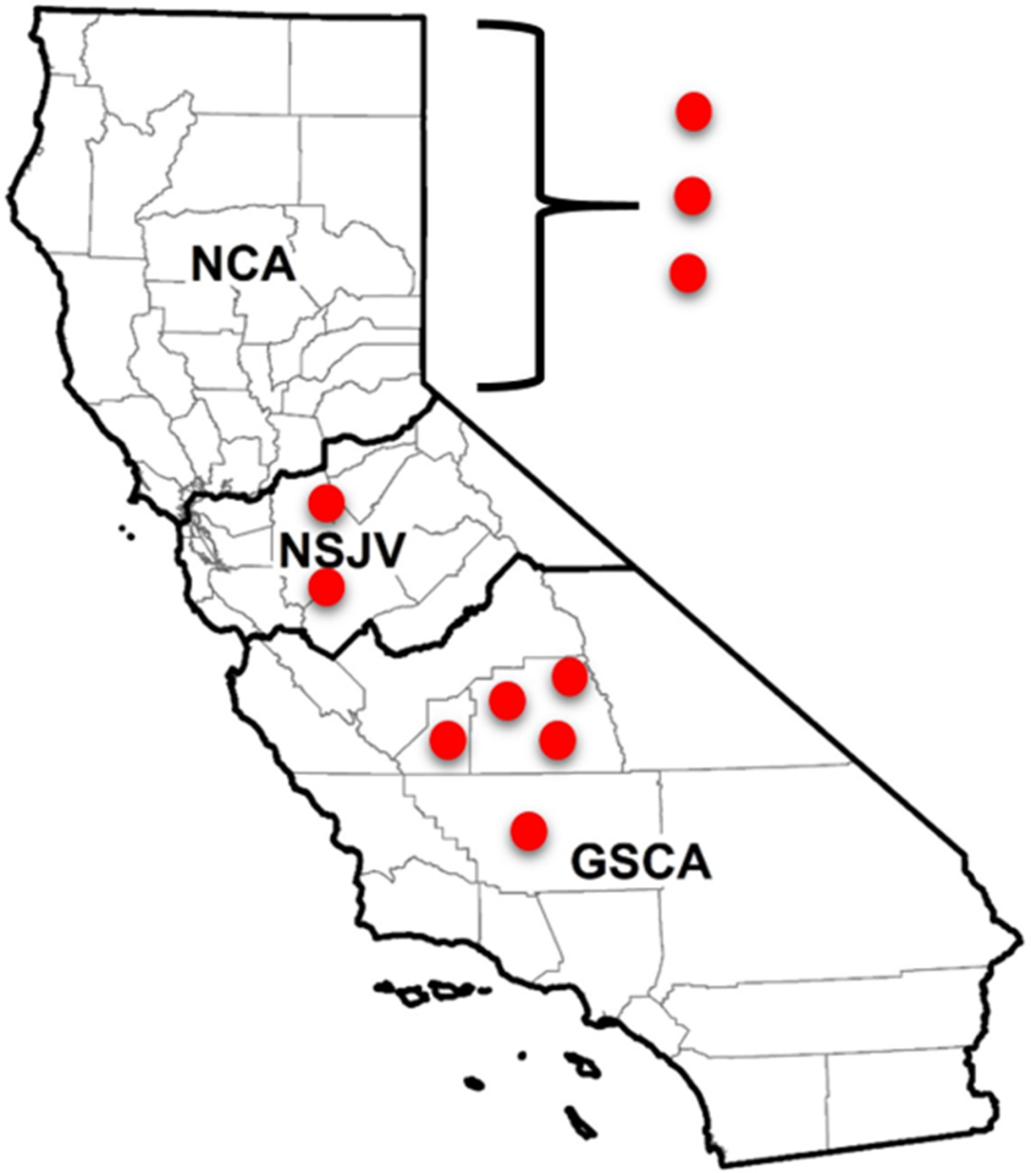
Locations of enrolled and sampled dairies for antimicrobial resistance testing in different regions of California. Locations of seven of 10 California dairies in Northern San Joaquin Valley (NSJV) and Greater Southern California (GSCA) where cohorts of adult cows were enrolled and sampled for antimicrobial resistance testing over Winter 2018 and Summer 2019. Locations of the three Northern California (NCA) dairies are censored to maintain confidentiality.

A total of 10 dairies were enrolled in the study and were distributed throughout the state’s three dairy regions: Northern California (NCA), Northern San Joaquin Valley (NSJV), and the Greater Southern California (GSCA). The three regions have distinct dairy infrastructure and management practices (Love et al., 2016; Karle et al., 2019), as well as distinct weather and climate conditions (Stull et al., 2008). Two of the study dairies were in Kern, two in Tulare, one in Kings and two in Stanislaus county. The remaining three dairies were in two counties in NCA; their county locations are withheld to maintain confidentiality (Fig. 1).

### Study design and cohorts

The current longitudinal study was conducted on the ten study dairies, each enrolling two cohorts of cows over two seasons. The first season’s cohort of cows was sampled over the fall and winter of 2018 (15 October 2018 to 25 March 2019; here onwards referred to as the winter cohort). The second season’s cohort of cows was sampled over the spring and summer of 2019 (4 March to 26 August 2019; here onwards referred to as the summer cohort). Each dairy was visited five times per cohort, every 4–5 weeks. A random sample of 12 cows per dairy per cohort were enrolled in the study before calving (close-up stage) and followed monthly thereafter for four consecutive time points post-calving, up to 120 days in milk (DIM). At the beginning of each cohort, we enrolled a stratified sample of cows based on the parity profile of the herd such that once the 12 selected cows had calved, they would represent their herd’s parity distribution of first, second and third or greater lactations. As a result, selection of cows was conducted using a random number generator (Excel; Microsoft Corp., Redmond, WA, USA) identifying the respective percent of the 12-cow cohort to be enrolled as either nulliparous, uniparous, or multiparous dams, approximately 2 weeks prior to their expected calving date. Enrolled cows at each farm were identified using neck collars, and individual fecal samples were collected at each visit, unless cows had been culled or died.

### Sample collection

Trained study personnel manually collected the fecal sample from the rectum of each cow using individual disposable sleeves and sterile lubricant. Feces were placed in a pre-labelled 50 mL sterile polypropylene container and transported or shipped on wet ice to the Dairy Epidemiology Lab at the Veterinary Medicine Teaching and Research Center, Tulare, CA. Samples were processed upon arrival or stored at 4 °C and processed within 24 h of collection. Samples collected from the three most distant northern dairies were shipped on ice overnight to the Dairy Epidemiology Lab and processed within 24 h of shipment. All samples from the remaining seven herds were transported and processed the same day.

### Bacteriological isolation and identification

#### Isolation of gram-negative fecal commensals

Fresh fecal samples were directly plated onto *E. coli* ChromoSelect agar with MUG (Sigma–Aldrich, St. Louis MO, USA) for *E. coli* isolation. Sterile cotton swab soaked in 1X Tris-HCl buffer was used to stab the fecal sample and spread onto the selective agar plate. The plates were incubated for 18–24 h at 44 °C and presumptive *E. coli* colonies were identified by the characteristic blue color and fluorescence under UV light illumination. Two discrete *E. coli* colonies from each sample were subcultured on tryptone soy agar with 5% sheep blood (Remel^™^, Lenexa, KS, USA) and incubated at 37 °C for a 24 h period. The two isolates were then tested for antimicrobial susceptibility. Presumptive *E. coli* colonies that had color variations inconsistent with *E. coli* on the specific ChromoSelect agar were confirmed by Gram staining and positive catalase test, Triple Sugar Iron (TSI) reaction resulting in A/A +− reaction, negative citrate and urea utilization tests, negative oxidase test and positive indole spot test.

#### Isolation of gram-positive fecal commensals

*Fresh fecal* samples were direct plating on Rapid Enterococci ChromoSelect agar (Sigma–Aldrich, St. Louis, MO, USA) following the same procedures as described for *E. coli* above. The plates were incubated for 18–24 h at 35 °C. Per the manufacturer provided information, ChromoSelect agar cannot differentiate between *Enterococcus* spp. and *Streptococcus* spp. by physical appearance (Manafi & Sommer, 1993). Hence, colonies isolated from the Rapid Enterococci ChromoSelect agar were here onwards referred to as *Enterococcus* spp./*Streptococcus* spp. (ES). ES were identified by the characteristic blue-green colonies. Two clearly defined, isolated colonies were picked from each individual fecal sample culture, streaked on sheep blood agar, and incubated at 37 °C for a 24 h period. The purified colonies were tested for antimicrobial susceptibility. Colonies that had color variation inconsistent with the specific ChromoSelect agar were confirmed by Gram staining, and negative catalase reaction.

### Antimicrobial susceptibility testing

Isolates were tested for antimicrobial susceptibility using the broth microdilution method (CLSI, 2018a). The Sensititre^™^ system (Thermo Fisher Scientific Inc., Waltham, MA, USA) was used to determine the minimum inhibitory concentration (MIC) of each isolate for the panel of drugs in the Sensititre^™^ Bovine BOPO7F Vet Antimicrobial Susceptibility Testing Plate (Thermo Scientific, Remel Inc., Lenexa, KS, USA). The Sensititre Bovine BOPO7F Plate was selected because it captured a broad range of antimicrobials including the latest antimicrobials approved for the treatment of bovine respiratory disease in cattle (gamithromycin and tildipirosin). Briefly, one to five suspect *E. coli* or ES colonies were resuspended in five mL of demineralized water (Thermo Scientific, Remel Inc., Lenexa, KS, USA) and the concentration adjusted to approximately 0.5 McFarland standard as measured by the Sensititre^™^ nephelometer. Subsequently, 10 μL (*E. coli*) or 30 μL (ES) of the 0.5 McFarland bacterial solution were added to 11 mL Mueller-Hinton broth (Thermo Scientific, Remel Inc., Lenexa, KS, USA), mixed by repeated inversion of the tube and 50 μL were inoculated into each well of the 96-well BOPO7F Vet plate using Sensititre^™^ Automated Inoculation Delivery System. One microliter of the inoculum broth from the positive control well of the plate was streaked on TSA w/5% Sheep Blood and incubated at 37 °C for 18–24 h to check for bacterial growth and colony purity. Inoculated MIC plates were sealed with the adhesive cover and incubated at 37 °C for 18–24 h. Plates that had contamination or no growth on corresponding SBA were not read and repeated. The MIC plates were read using Sensititre^™^ Vizion Digital MIC Viewing System and Thermo Scientific Sensititre SWIN Software System. During the first 2 weeks of reading the MIC plates, the quality control measures were conducted using five control strains including *E. coli ATCC 35218, E. coli ATCC 25922, Enterococcus faecalis ATCC 29212, Strep. pneumoniae ATCC 49619, Histophilus somni 700025*. Quality control measures were then run weekly using only three control strains: *E. coli ATCC 35218, E. coli ATCC 25922, Enterococcus faecalis ATCC 29212*.

The MIC values were recorded as the lowest concentration of antimicrobial drug that inhibited the growth of bacteria. The panel of drugs for which isolates were tested included 19 antibiotics comprised of ampicillin, clindamycin, danofloxacin, enrofloxacin, florfenicol, gamithromycin, gentamicin, neomycin, penicillin, sulphadimethoxine, spectinomycin, trimethoprim-sulfamethoxazole, tetracycline, tiamulin, tilmicosin, tildipirosin, tulathromycin, tylosin tartrate and ceftiofur. Interpretations of antibiotic resistance followed MIC breakpoints set by the Clinical and Laboratory Standards Institute (CLSI) if available; otherwise, MIC breakpoints suggested by other publications were used (See Tables S1 and S2). Isolates were classified as susceptible or resistant (intermediate isolates were classified as resistant).

A stratified random sample of 200 isolates from the study repository, stratified by species, region and season cohort were identified after testing for antimicrobial susceptibility and submitted for species confirmation using MALDI-ToF MS at California Animal Health and Food Safety Laboratory (Tulare, CA, USA), and 16S rRNA partial gene sequencing at UC Berkeley DNA Sequencing Facility (Berkeley, CA USA). Based on the species confirmed, breakpoints were identified as illustrated in Tables S1 and S2. For ES isolates, only ampicillin and florfenicol had identical breakpoints for both *Enterococcus* spp. and *Streptococcus* spp. For the remaining drugs (penicillin, tetracycline, tiamulin, gamithromycin, tilmicosin, tildipirosin, tulathromycin and tylosin) the *Enterococcus* spp. breakpoints were used to determine AMR status of the ES isolates.

*Enterobacterales* are intrinsically resistant to clindamycin and macrolides (CLSI, 2018b) and penicillin (Burman, Nordström & Boman, 1968; Magiorakos et al., 2012; Tadesse et al., 2012; Van Hoek et al., 2011). Therefore, clindamycin, macrolides and penicillin were excluded from the interpretation of antibiotic resistance and calculation of multidrug resistance for *E. coli*. An *E. coli* isolate was classified as multidrug-resistant (MDR) when it was resistant to at least one drug in three or more antimicrobial classes: penicillins, cephalosporins, fluoroquinolones, amphenicols, tetracyclines, aminoglycosides and folate pathway antagonists. Similarly, *Enterococcus* spp. are intrinsically resistant to cephalosporins, lincosamides, aminoglycosides, fluoroquinolones, sulfonamides and trimethoprim-sulfamethoxazole (Hollenbeck & Rice, 2012) and drugs in these classes were excluded from analysis and the interpretation of antibiotic resistance or multidrug resistance for *Enterococcus* spp. An ES isolate was classified as MDR when it was resistant to at least one drug in three or more antimicrobial classes: penicillins, amphenicols, tetracyclines, pleuromutilins and macrolides.

### Statistical analysis

Data were entered in spreadsheets before being housed along with sample collection information in a relational database (Microsoft Access, Microsoft Corp., Redmond WA). Statistical analyses were conducted using Stata versions 15 and 16 (Stata Corp. LLC, College Station, TX, USA). Proportions with respective standard errors (SE) were computed for categorical variables, while means and respective SE were computed for continuous variables. The proportions of resistant isolates and associated 95% CI were reported for each antimicrobial drug across season, region, and sampling point (close-up, and 30, 60, 90 and 120 DIM). The frequency and proportion of MDR *E. coli* and ES isolates were calculated over season, region and sampling point. The hierarchical cluster analysis was performed by Ward’s clustering method with squared Euclidean distance for binary AMR with categorical factors (Berge, Atwill & Sischo, 2003). The identified clusters were then described based on study cohort, region, sampling point, calving status and AMR for each antibiotic. Differences between clusters were compared using their 95% CI coverage.

## Results

### Descriptive results of study herd management practices

Enrolled study herds included five dairies in GSCA, two dairies in NSJV, and three dairies in NCA (Table 1). Holstein was the most common breed among all farms, followed by crossbred and Jersey breeds. The mean ± SE number of milking cows in enrolled dairies was 1,605.5 ± 462.2. The average somatic cell count (SCC) for the ten dairy herds was found to be approximately 140,000 ± 17,950 cells per ml with a rolling herd average milk production of 11,390 ± 530.57 Kg/cow.

**Table 1:** Descriptive data for ten California dairy herds enrolled in a longitudinal study to determine the antimicrobial resistance in *Escherichia coli* and *Enterococcus* spp./ *Streptococcus* spp. isolated from fecal samples of dairy cows.

Herd	Location	Mean milking herd size	RHA, Kg/cow^1^	Herd breed, (%)^2^	Use of Antibiotic at dry-off^3^
1	Greater Southern CA	2,700	8,940	J (100)	Yes, Blanket treatment
2		870	12,701	H (100)	Yes, Blanket treatment
3		325	10,886	H (40), X (20), J (40)	Yes, Blanket treatment
4		2,500	13,245	H (100)	Yes, Blanket treatment
5		5,000	13,245	H (100)	Yes, Blanket treatment
6	Northern San Joaquin Valley CA	1,250	13,154	X (100)	Yes, Blanket treatment
7		1,000	11,340	H (100)	Yes, Blanket treatment
8	Northern CA	1,600	11,340	H (100)	Yes, Blanket treatment
9		680	9,979	H (90), X (10)	No Antibiotic treatment
10		130	9,072	H (100)	No Antibiotic treatment
Mean ± SE		1,605.5 ± 462.21	11,390 ± 530.57		

**Notes:**

1 Rolling herd average defined as the mean milk produced per milking cow in the herd during the previous year.

2 Holstein (H), Crossbred (X), and Jersey (J) breeds.

3 Blanket treatment defined as treat all dry-cows with intramammary dry-cow antibiotics and/or internal teat sealant.

A summary characteristic of the study herds and management practices is presented in Table 1. Three farms from GSCA and one farm from NSJV administered only intramammary antibiotics at dry-off, while two farms from GSCA, and one farm each from NSJV and NCA administered both antibiotics and internal teat sealant at dry-off to all lactating cows (blanket dry-cow therapy, BDCT). The remaining two farms from NCA did not treat their cows at dry-off with antibiotics. Among the farms practicing BDCT, four farms used ceftiofur hydrochloride, three farms used cephaprin benzathine, and one farm used penicillin G procaine/dihydrostreptomycin combination antibiotic. The study dairies’ first choice antibiotic for treatment of clinical mastitis was ceftiofur hydrochloride (five farms) and cephaprin sodium (three farms), while two farms in NCA did not treat clinical mastitis with antibiotics. The mean number of cows treated for mastitis over the ten farms was 39 ± 13 cows/per month. Eight out of 10 farms vaccinated their cows against coliform mastitis.

In seven herds, the lactating dairy cows were housed in free stall barns bedded with dried manure solids, while dry and hospital cows were housed in open-lot pens with concrete flush lanes in the feed alley. All seven of these dairies used recycled lagoon water to flush manure from the lanes. In addition, one herd utilized a pasture system, one herd utilized mixed pasture and freestall barns, and one herd housed all cows in open-lot pens.

### Bacterial identification

**Figure 2:**
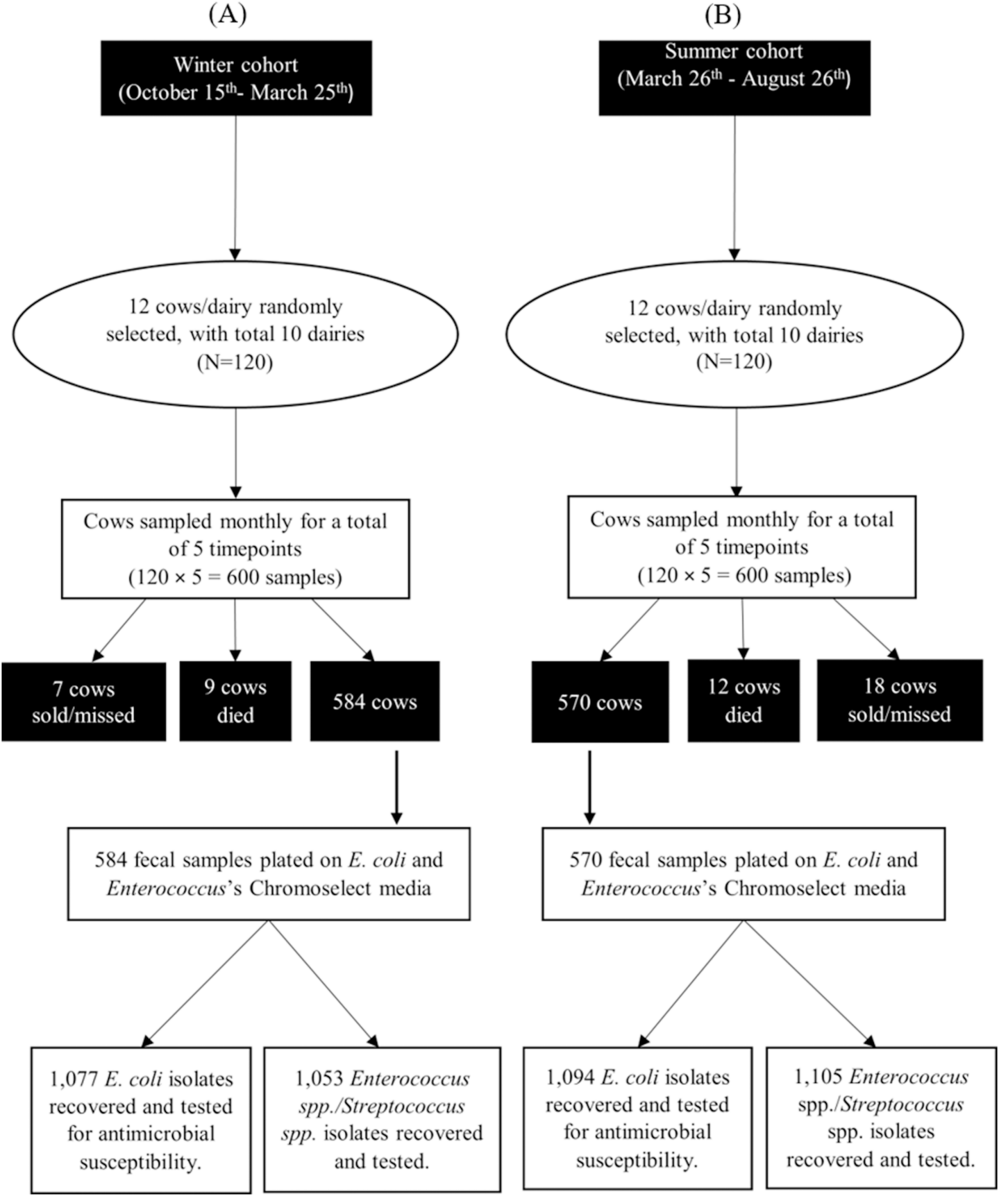
Flow diagram summarizing the number of fecal commensal bacterial isolates from winter (A) and summer (B) cohorts of cows on 10 California dairies.

A total of 584 and 570 fecal samples were collected from dairy cows during winter and summer seasons, respectively. From these samples 2,171 *E. coli* isolates (1,077 in winter and 1,094 in summer) and 2,158 ES isolates (1,053 in winter and 1,105 in summer) were identified (Fig. 2). Among these isolates, 2,169 *E. coli* and 2,157 ES isolates were tested for antimicrobial susceptibility. Some fecal samples failed to yield either *E. coli* (68 samples) or ES (75 samples) after three culture attempts and were, therefore, excluded. Results of MALDI-ToF MS and genotyping by 16S rRNA partial gene sequencing for the stratified random samples of 200 isolates were in perfect agreement with exception of a single ChromoSelect-confirmed *Enterococcus* spp. isolate that was unidentified using MALDI-ToF but confirmed as *Streptococcus equinus* using 16S rRNA partial gene sequencing. At the species level, only two out of the 103 ChromoSelect-confirmed *E. coli* isolates were *Salmonella* sp., the remaining were *E. coli* (98.05%). For the 97 ChromoSelect-confirmed *Enterococcus* spp. isolates, 54 were *Streptococcus* spp., the remaining 43 were *Enterococcus* spp. (44.32%).

### AMR of isolates

Antimicrobial susceptibility results of *E. coli* isolates are summarized in Table 2. Our results indicated that a low proportion of *E. coli* isolates were resistant to ampicillin, ceftiofur, danofloxacin, enrofloxacin, gentamicin, neomycin, spectinomycin, trimethoprim-sulfamethoxazole and tulathromycin. However, a high rate of resistance among all tested antimicrobial drugs was recorded for tylosin, tilmicosin and tiamulin. Among ES, a low proportion of the isolates were resistant to penicillin (0.18% ± 0.09), ampicillin (0.23% ± 0.10), tylosin (3.19% ± 0.37) and tulathromycin (7.64% ± 0.57) (Table 3). Approximately half of the ES isolates were resistant to tildipirosin (50.18% ± 1.10), tilmicosin (47.91% ± 1.10) and florfenicol (46.54% ± 1.10). The frequency of ES resistance to gamithromycin and tetracycline was 11.54% ± 0.68 and 15.25% ± 0.70% respectively (Table 3).

**Table 2:** MIC distribution frequency of *Escherichia coli* isolated from bovine fecal samples collected over winter and summer cohorts from 2018–2019 (*n* = 2,169).

Antimicrobial agent minimum inhibitory concentration (μg/mL)	Number and (percentage) of isolates with MICs (µg/mL)^1^													Resistance %^2^	MIC 50	MIC 90
	0.12	0.25	0.5	1	2	4	8	16	32	64	128	256	512			
Ampicillin		2 (0.09)	18 (0.83)	127 (5.86)	977 (45.00)	995 (45.83)	27 (1.24)	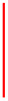 2 (0.09)	21 (0.97)					1.10	2	4
Ceftiofur		1,037 (47.81)	1,032 (47.58)	52 (2.40)	6 (0.28)	2 (0.09)	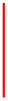 5 (0.23)	35 (1.61)						1.93	0.5	0.5
Danofloxacin	2,063 (95.11)	19 (0.88)	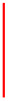 5 (0.23)	25 (1.15)	57 (2.63)									4.01	0.12	0.12
Enrofloxacin	2,076 (95.71)	21 (0.97)	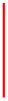 6 (0.28)	6 (0.28)	11 (0.51)	49 (2.26)								3.31	0.12	0.12
Florfenicol		0 -	0 -	10 (0.46)	352 (16.23)	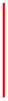 1,360 (62.70)	329 (15.17)	118 (5.44)						83.31	4	8
Gamithromycin				6 (0.28)	48 (2.21)	509 (23.47)	1,365 (62.93)	241 (11.11)						-	8	16
Gentamicin				2,093 (96.50)	61 (2.81)	8 (0.37)	6 (0.28)	1 (0.05)						0.32	1	1
Neomycin						2,134 (98.30)	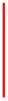 31 (1.43)	1 (0.05)	1 (0.05)	2 (0.09)				1.61	4	4
Spectinomycin							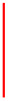 136 (6.27)	1,740 (80.22)	183 (8.44)	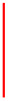 40 (1.84)	70 (3.23)			5.10	16	32
Sulphadimethoxine												1,465 (67.54)	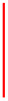 704 (32.46)	32.45	256	512
Tetracycline			18 (0.83)	884 (40.76)	902 (41.59)	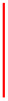 102 (4.70)	44 (2.03)	219 (10.10)						16.82	2	16
Tiamulin			2 (0.09)	0 -	1 (0.05)	0 -	0 -	20 (0.92)	171 (7.89)	1,973 (91.05)					64	64
Tildipirosin				29 (1.34)	282 (13.00)	1,413 (65.15)	355 (16.37)	32 (1.48)	58 (2.67)						4	8
Tilmicosin					1 (0.05)	0 -	0 -	17 (0.78)	2,147 (99.08)						32	32
Trimethoprim-sulfamethoxazole					2,072 (95.53)	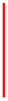 97 (4.47)								4.47	2	2
Tulathromycin							1,987 (91.61)	135 (6.22)	13 (0.60)	6 (0.28)	28 (1.29)			2.16	8	8
Tylosin			1 (0.05)	0 -	0 -	1 (0.05)	0 -	0 -	4 (0.18)	2,162 (99.72)				99.86	64	64

**Notes:**

1 Vertical red line indicate resistance breakpoints.

2 Resistance breakpoints are missing for macrolides and pleuromutilin.

**Table 3:** Frequency of *Enterococcus* spp./ *Streptococcus* spp. isolated from bovine fecal samples by antimicrobial resistance as measured by minimum inhibitory concentration (µg/ml). Fecal samples were collected from 10 California dairies over two ccohorts from 2018–2019 (*n* = 2,157).

Antimicrobial agent	Number and percentage of isolates with MICs (ug/ml)^1^											Resistant %	MIC 50	MIC 90
	0.12	0.25	0.5	1	2	4	8	16	32	64	128			
Ampicillin		1,393 (64.58)	400 (18.54)	301 (13.95)	37 (1.72)	11 (0.51)	10 (0.46)	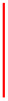 1 (0.05)	4 (0.19)			0.23	0.25	1
Florfenicol		27 (1.25)	15 (0.70)	542 (25.13)	569 (26.38)	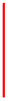 813 (37.69)	64 (2.97)	127 (5.89)				46.54	2	8
Gamithromycin^2^				1,769 (82.01)	58 (2.69)	81 (3.76)	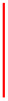 111 (5.15)	138 (6.40)				11.54	1	8
Penicillin^2^	1,236 (57.30)	61 (2.83)	222 (10.29)	270 (12.52)	231 (10.71)	116 (5.38)	17 (0.79)	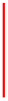 4 (0.19)				0.18	0.12	2
Tetracycline^2^			1,482 (68.71)	306 (14.19)	40 (1.85)	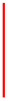 38 (1.76)	23 (1.07)	268 (12.42)				15.25	0.5	16
Tiamulin^2^			984 (45.62)	122 (5.66)	89 (4.13)	16 (0.74)	22 (1.02)	10 (0.64)	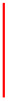 38 (1.76)	876 (40.61)		42.37	1	64
Tildipirosin^2^				923 (42.81)	116 (5.38)	35 (1.62)	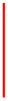 37 (1.72)	122 (5.66)	923 (42.81)			50.18	8	32
Tilmicosin^2^					1,064 (49.35)	27 (1.25)	32 (1.48)	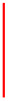 311 (14.42)	722 (33.49)			47.91	4	32
Tulathromycin^2^							1,854 (85.95)	138 (6.40)	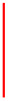 81 (3.76)	13 (0.60)	71 (3.29)	7.64	8	16
Tylosin^2^			1,051 (48.73)	82 (3.80)	512 (23.74)	369 (17.11)	74 (3.43)	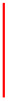 16 (0.74)	1 (0.05)	52 (2.41)		3.19	1	4

**Notes:**

1 Vertical red lines indicate resistance breakpoints.

2 Antimicrobial resistance estimates are based on *Enterococcus* spp. breakpoints. Due to the difference in breakpoints for *Enterococcus* spp. and *Streptococcus* spp., estimates should be interpreted with caution due to the potential for underestimating resistance.

### Prevalence of AMR across seasons

**Figure 3:**
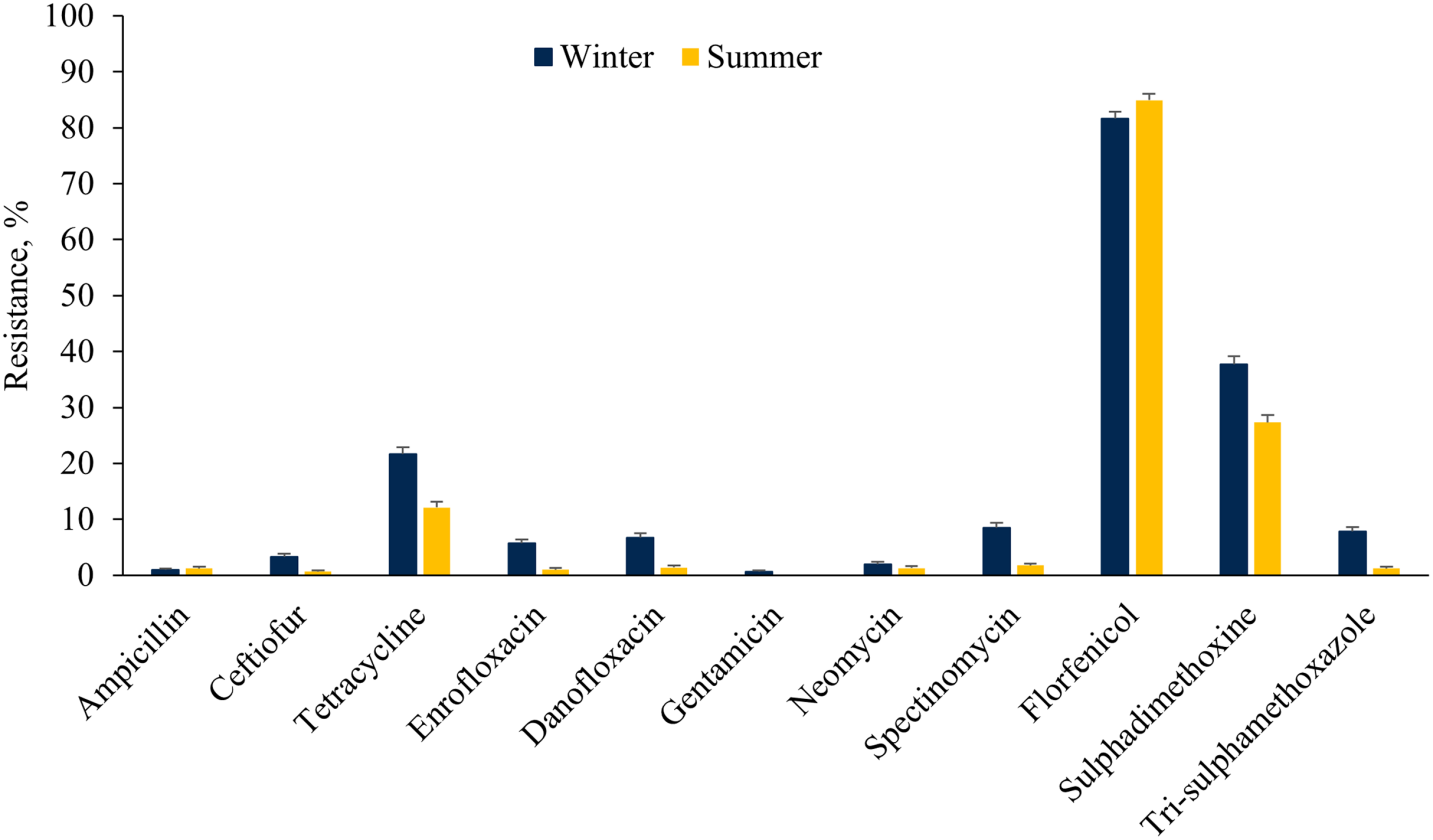
Resistance of *Escherichia coli* isolated from fecal samples of California dairy cows over winter and summer cohorts from 2018 to 2019.

**Figure 4:**
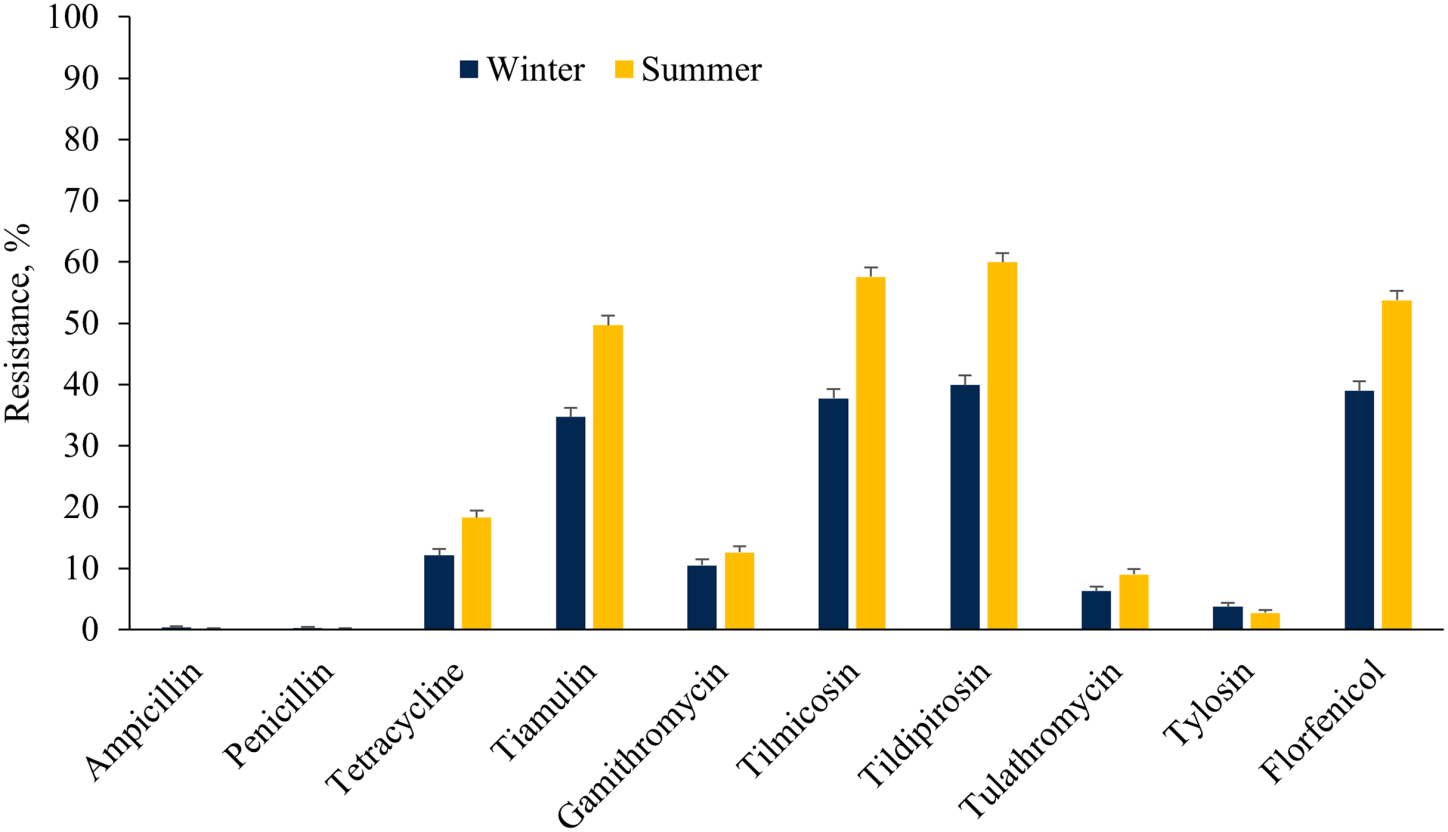
Resistance of *Enterococcus* spp./*Streptococcus* spp. isolated from fecal samples of California dairy cows over winter and summer cohorts from 2018 to 2019.

The *E. coli* isolates showed higher resistance to ceftiofur, tetracycline, fluoroquinolones, aminoglycosides (except neomycin), sulphadimethoxine and trimethoprim-sulfamethoxazole in the winter as compared to the summer cohort (Fig. 3). However, a higher proportion of *E. coli* isolates were resistant to florfenicol during summer as compared to winter. On the other hand, a higher proportion of ES isolates were resistant to tetracycline, tiamulin, tilmicosin, tildipirosin, tulathromycin and florfenicol during summer rather than the winter season (Fig. 4).

### Regional distribution of AMR

**Figure 5:**
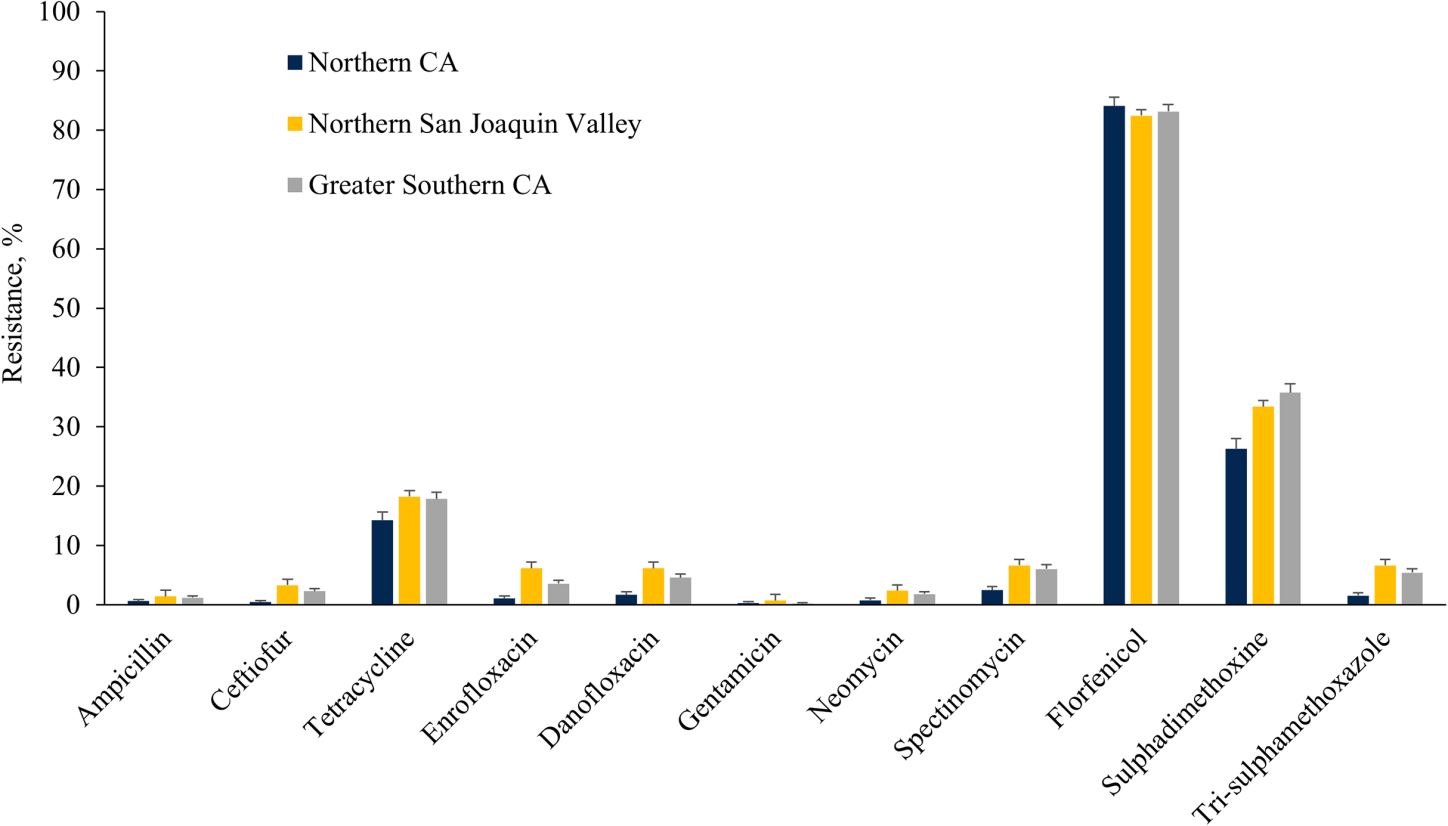
Resistance of *Escherichia coli* isolates originated from fecal samples of California dairy cows in different regions of CA over two cohorts from 2018 to 2019.

**Figure 6:**
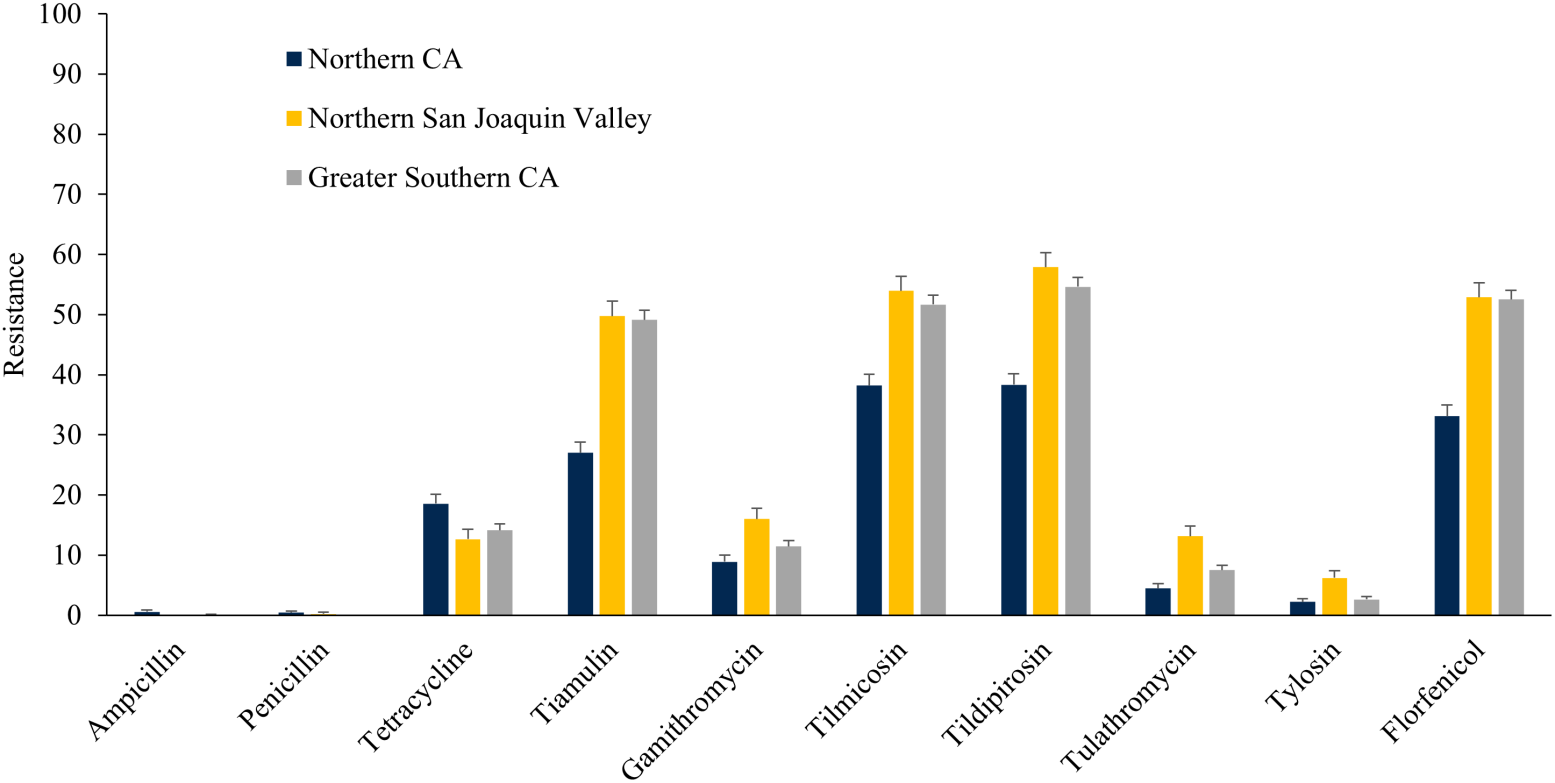
Resistance of *Enterococcus spp./Streptococcus spp*. isolates originated from fecal samples of California dairy cows in different regions of CA over two cohorts from 2018 to 2019.

The *E. coli* isolates obtained throughout the study from NSJV and GSCA showed greater resistance to ceftiofur, danofloxacin, spectinomycin, sulphadimethoxine, and trimethoprim-sulfamethoxazole compared to isolates from NCA. However, higher resistance against tylosin was observed in *E. coli* isolates from NCA in comparison to NSJV and GSCA (Fig. 5). Similarly, the ES isolates obtained throughout the study from NSJV and/or GSCA showed greater resistance to tiamulin, macrolides and florfenicol in comparison to isolates from NCA. However, ES isolates from NCA showed higher resistance to penicillins (ampicillin and penicillin), and tetracycline in comparison to isolates obtained from NSJV or GSCA (Fig. 6).

*Escherichia coli* isolated from cows in NCA during the winter cohort had lower resistance to ceftiofur, fluoroquinolones, spectinomycin, sulphadimethoxine, and trimethoprim-sulfamethoxazole compared to isolates from cows in NSJV and GSCA (Table S3). Regional distributions of *E. coli* resistance during the summer cohort are summarized in Table S4.

During the winter cohort, the ES isolates obtained from fecal samples of cows in NCA were more susceptible to pleuromutilins (tiamulin), macrolides and amphenicols (florfenicol), but were highly resistant to penicillin as compared to isolates from NSJV or GSCA (Table S5). Furthermore, the resistance of ES isolates obtained from cows in NCA were lower for tiamulin, tilmicosin, tildipirosin and florfenicol in comparison to isolates obtained from NSJV or GSCA during the summer cohort (Table S6).

### AMR profile across sampling points

**Figure 7:**
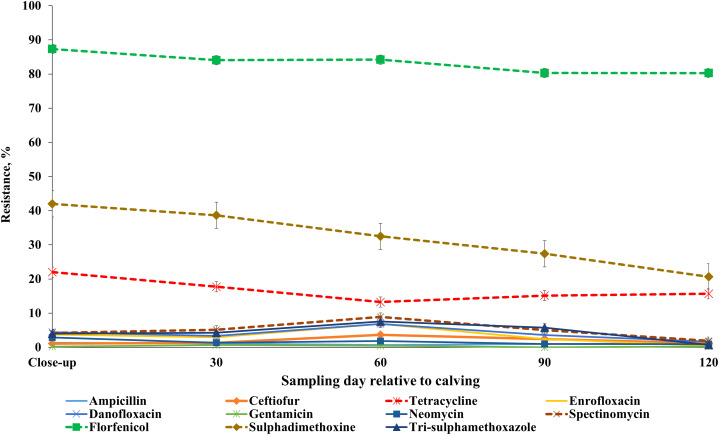
Antimicrobial resistance profiles of fecal *E. coli* from adult dairy cows over production stage staring with late pregnancy non-lactating cows (close-up) to 120 days post calving over two cohorts from 2018 to 2019.

**Figure 8:**
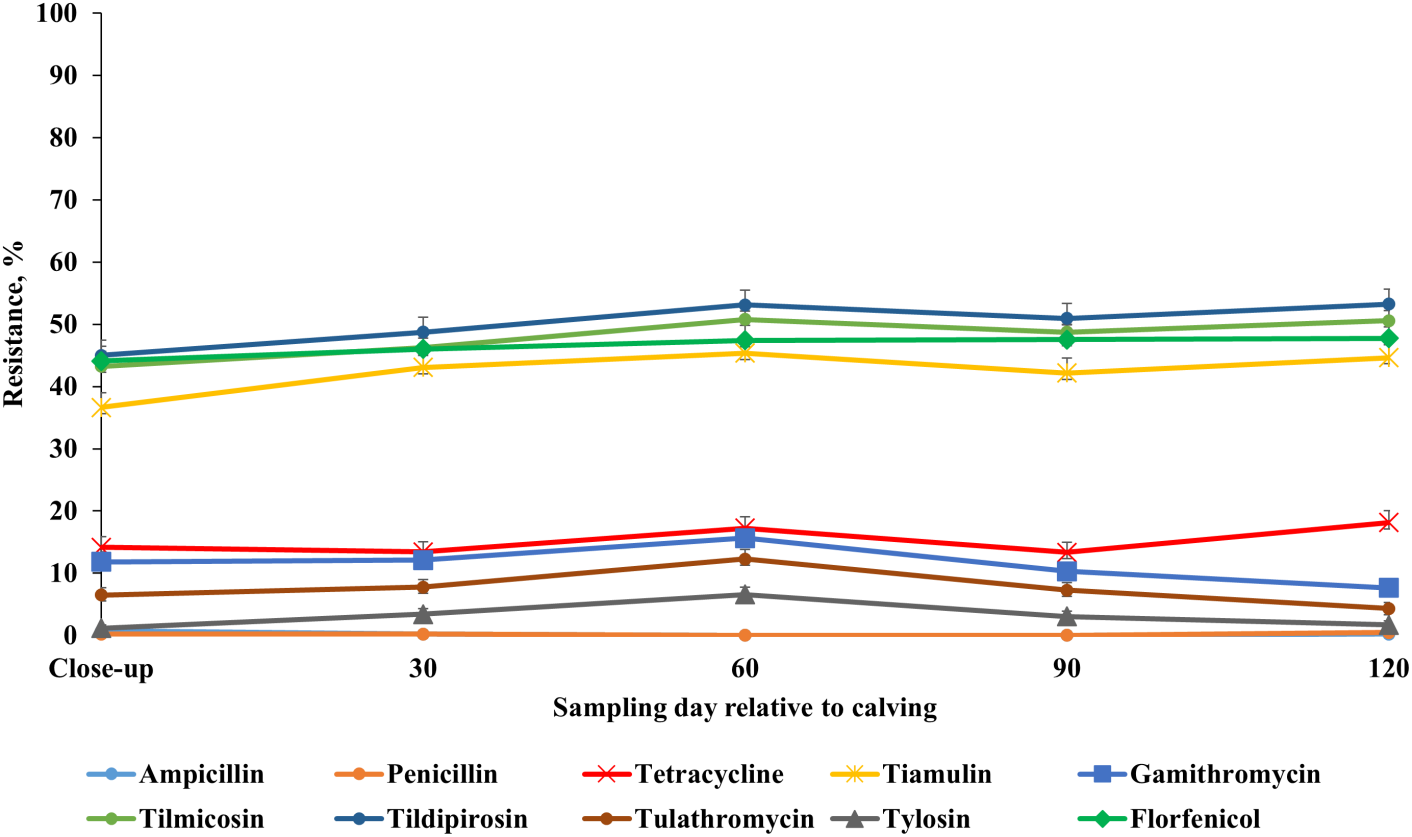
Antimicrobial resistance profile of fecal *Enterococcus* spp./*Streptococcus* spp. from adult dairy cows from close-up to 120 days post-calving over two seasonal cohorts from 2018 to 2019.

Our results showed that the resistance of *E. coli* isolates to ceftiofur, enrofloxacin, danofloxacin, spectinomycin and trimethoprim-sulfamethoxazole was higher at 60 DIM in comparison to other sampling points. *E. coli* resistance to tetracycline, tildipirosin, florfenicol, and sulphadimethoxine was higher at close-up in comparison to all the subsequent sample periods post-calving (Fig. 7; Table S7). Detailed information regarding the prevalence of resistance in *E. coli* isolates across all sampling points during the winter and summer cohorts are presented in Tables S8 and S9. Also, a higher proportion of ES isolates were resistant to tiamulin, gamithromycin, tulathromycin and tylosin at 60 DIM in comparison to other sampling points (Fig. 8; Table S10). The prevalence of AMR in ES isolates across sampling points during the winter and summer cohorts are presented in Tables S11 and S12.

### Multidrug resistance in *E. coli* isolates

Multidrug resistance was observed in 307 (14.14%) *E. coli* isolates, while a similar proportion (13.19%) were pan-susceptible to the antimicrobial drugs tested in our study. A majority of the 2,160 *E. coli* isolates tested were resistant to one antimicrobial drug class (48.87%) and another 23.88% of isolates were resistant to two antimicrobial drugs classes. Resistance against six antimicrobial classes was observed in seven out of 2,169 isolates.

*Escherichia coli* isolates recovered during the winter season showed a higher proportion of MDR than those isolated during the summer season (Table 4). Higher prevalence of MDR *E. coli* occurred in GSCA and NSJA in comparison to NCA (Table 4). The prevalence of MDR *E. coli* was higher in fecal samples collected from dairy cows during the close-up and on 30 and 60 DIM in comparison to those collected at 90 and 120 DIM (Table 4).

**Table 4:** Frequency (percentage) of antimicrobial resistance observed for *Escherichia coli* isolated from fecal samples of dairy cattle in California^1^.

Variable	No. of isolates	Fully susceptible	Resistance to one antimicrobial drug class	Resistance to two antimicrobials drug classes	MDR^2^
Season					
Winter	1,077	153 (14.21)	462 (42.89)	243 (22.60)	219 (20.33)
Summer	1,094	133 (12.34)	598 (54.66)	275 (25.13)	88 (8.04)
Region					
Northern CA	654	85 (12.99)	366 (55.95)	137 (20.94)	66 (10.10)
Northern San Joaquin Valley	422	56 (13.27)	206 (48.81)	87 (21.10)	73(17.29)
Greater Southern CA	1,095	145 (13.26)	488 (44.64)	294 (26.89)	168 (15.34)
Sampling point, DIM^3^					
Close-up	452	42 (9.29)	191 (42.25)	130 (28.76)	89 (19.69)
30	445	50 (11.23)	212 (47.64)	118 (26.51)	65 (14.61)
60	437	56 (12.81)	216 (49.42)	100 (22.88)	65 (14.87)
90	416	66 (15.86)	216 (52.10)	85 (20.43)	49 (11.77)
120	421	72 (17.10)	225 (53.44)	85 (20.19)	39 (9.26)

**Notes:**

1 proportion and frequencies based on antimicrobial drug class and hence resistance to one, two, or more classes may not be in decreasing frequencies.

2 MDR, the resistance of a bacterial isolate to antibiotics belonging to at least three different classes is defined as multidrug resistance.

3 DIM=Days in milk

### Multidrug resistance in *Enterococcus* spp./*Streptococcus* spp. isolates

The distribution of MDR ES isolates from dairy cattle in CA is shown in Table 5. Approximately 38.64% (834 of 2,157) of ES isolates were defined as multidrug resistant. A similar proportion, (804 of 2,157; 37.25%) of ES isolates were fully susceptible to the five antimicrobial drug classes tested in this study. The ES isolated from cows during the summer cohort showed higher proportions of MDR than the isolates obtained from cows during the winter cohort. The prevalence of MDR ES in fecal samples collected from dairy cows was lower in NCA (24.92% ± 1.66) in comparison to NSJA (48.10% ± 2.44) and GSCA (43.62% ± 1.51).

**Table 5:** Frequency (percentage) of antimicrobial resistance observed for *Enterococcus* spp./ *Streptococcus* spp. isolated from fecal samples of dairy cattle in CA^1^^,^^2^.

Variable	No. of isolates	Fully susceptible	Resistance to one antimicrobial drug class	Resistance to two antimicrobials drug class	MDR^3^
Season					
Winter	1,053	483 (45.86)	135 (12.82)	111 (10.54)	324 (30.76)
Summer	1,105	321 (29.04)	128 (11.58)	146 (13.21)	510 (46.15)
Region					
Northern CA	674	298 (44.21)	131 (19.43)	77 (11.42)	168 (24.92)
Northern San Joaquin Valley	418	155 (37.10)	24 (5.74)	38 (9.10)	201 (48.10)
Greater Southern CA	1,066	351 (32.92)	108 (10.13)	142 (13.32)	465 (43.62)
Sampling point, DIM^4^					
Close-up	432	187 (43.28)	41 (9.49)	49 (11.34)	153 (35.42)
30	439	168 (38.26)	57 (12.98)	47 (10.71)	167 (38.04)
60	441	150 (34.01)	56 (12.69)	56 (12.69)	179 (40.58)
90	427	161 (37.70)	48 (11.26)	54 (12.67)	163 (38.41)
120	419	138 (32.93)	61 (14.55)	51 (12.71)	169 (40.33)

**Notes:**

1 Proportion and frequencies based on antimicrobial drug class and hence resistance to one, two, or more classes may not be in decreasing frequencies.

2 Antimicrobial resistance estimates are based on *Enterococcus* spp. breakpoints. Due to the difference in breakpoints for *Enterococcus* spp. and *Streptococcus* spp., estimates should be interpreted with caution due to the potential for underestimating resistance.

3 MDR, the resistance of a bacterial isolate to antimicrobial drug belonging to at least three different classes is defined as multidrug resistance.

4 DIM, days in milk.

### Hierarchical clustering of *E. coli* and *Enterococcus* spp./*Streptococcus* spp. isolates

The *E. coli* AMR cluster characteristics and the number of isolates belonging to each cluster are shown in Table 6. The cut-off was set at two clusters to describe the isolates’ AMR phenotype over the study period. We found that two clusters better described the study isolates’ distinct clustering after also evaluating the separation into 3, 4 and 5 clusters. The frequency distribution of isolates from GSC was significantly higher in cluster 1 as compared to cluster 2; conversely, the distribution of isolates from NCA was greater in Cluster 2 as compared to cluster 1. A significantly higher frequency of resistant *E. coli* isolates from the winter cohort was observed in cluster 1 compared to cluster 2. The frequency of resistance in *E. coli* isolates from close-up cows was higher in cluster 1 compared to cluster 2, however the frequency of resistant *E. coli* isolates from cows at 90 and 120 DIM was higher in cluster 2 compared to cluster 1. *E. coli* isolates from cluster 1 showed significantly higher frequency of resistance to all antibiotics in comparison to isolates from cluster 2 (Table 6).

**Table 6:** Description of two typologies identified using Hierarchical clustering and allocation of *E. coli* isolates..

Characteristics	Cluster 1	Cluster 2
	*N* = 825 (%)	*N* = 1,344 (%)
Region		
Northern California	24.60	33.55
Northern San Joaquin Valley	19.63	19.34
Greater Southern CA	55.75	47.10
Cohort		
Winter	55.87	45.68
Summer	44.12	54.31
Sampling point, DIM		
Close-up	26.30	17.33
30	22.18	19.49
60	20.12	20.16
90	16.36	21.10
120	15.03	22.10
Calving status		
Before calving	26.30	17.33
After calving	73.69	82.66
Antibiotic Resistance		
Ampicillin	2.78	0.00
Ceftiofur	5.10	0.00
Danofloxacin	9.93	0.37
Enrofloxacin	8.72	0.00
Florfenicol	98.66	73.88
Tetracycline	42.66	0.96
Gentamicin	0.72	0.07
Neomycin	3.75	0.29
Spectinomycin	13.21	0.07
Sulphadimethoxine	80.36	3.10
Trimethoprim-sulfamethoxazole	11.51	0.14
*E. coli* MDR	100	0.00

The ES AMR cluster characteristics and the number of isolates belonging to each cluster are shown in Table 7. No seasonal effect was detected by clustering ES isolates. The ES isolates from cluster 1 showed higher frequency of resistance during the sampling period from close-up and 30 and 60 DIM in comparison to isolates from cluster 2 (Table 7). In contrast, no resistant ES isolates were identified at the 90 and 120 DIM in cluster 1. A significantly higher proportion of isolates from cluster 1 showed resistance to gamithromycin, tulathromycin, and tylosin in comparison to isolates captured by cluster 2.

**Table 7:** Description of two typologies identified using hierarchal clustering and allocation of *Enterococcus* spp. and *Streptococcus* spp. (ES) isolates.

Characteristics	Cluster 1	Cluster 2
	*N* = 1,296(%)	*N* = 861 (%)
Region		
Northern California	31.32	31.01
Northern San Joaquin Valley	19.21	19.62
Greater Southern CA	49.45	49.36
Cohort		
Winter	48.22	49.59
Summer	51.77	50.40
Sampling point, DIM		
Close-up	32.10	1.72
30	33.87	0.00
60	34.03	0.00
90	0.00	49.56
120	0.00	48.66
Calving status		
Before calving	32.10	1.74
After calving	67.90	98.25
Antibiotic Resistance		
Ampicillin	0.31	0.11
Penicillin	0.15	0.23
Florfenicol	45.52	48.10
Tetracycline	14.42	16.50
Tiamulin	42.01	42.97
Gamithromycin	13.11	9.20
Tulathromycin	8.72	6.03
Tilmicosin	46.37	50.20
Tildipirosin	48.62	52.49
Tylosin	3.78	2.32
*ES* MDR	37.88	39.72

### Phenotype resistance pattern of *E. coli* and *Enterococcus* spp./*Streptococcus* spp. isolates

The major phenotypic multidrug resistance patterns in fecal *E. coli* isolated from dairy cattle in our study were tetracycline-florfenicol-sulphadimethoxine (6.82%, Table 8). While the most common phenotypic resistance pattern observed for ES was tilmicosin-tildipirosin-tiamulin-florfenicol (22.01%, Table 9).

**Table 8:** Phenotype resistance pattern in *Escherichia coli* isolated from dairy cattle in different regions in California.

Resistance patterns^1^	No of isolates	Percent
No resistance	286	13.20
Flo	995	45.8
FloSdim	352	16.2
TetraFloSdim	148	6.82
TetraFlo	115	5.3
Sdim	39	1.7
CeftEnrDanSpecFloSdimTrisul	15	0.6
SpecFlo	15	0.6
Tetra	13	0.6
FloSdimTrisul	12	0.5
TetraFloSdimTrisul	10	0.5
EnrDanSpecFloSdimTrisul	8	0.3
NeoFlo	8	0.3
TetraEnrDanSpecFloSdimTrisul	8	0.3
NeoFloSdim	6	0.2
SpecFloSdim	6	0.2
Dan	5	0.2
DanFlo	5	0.2
TetraDanFloSdim	5	0.2
TetraSpecFloSdimTrisul	5	0.2
Other combinations	Less than 5 isolates	

**Note:**

1 Flo, Florfenicol; Sdim, Sulphadimethoxine; Tetra, Tetracycline; Ceft, Ceftioufor; Enr, Enerofloxacine; Dan, Danofloxacin; Spec, Spectinomycine; Trisul, Trimethoprim-sulfamethoxazole.

**Table 9:** Phenotype resistance pattern in fecal *Enterococcus* spp./*Streptococcus* spp. isolated from dairy cattle in different regions in California.

Pattern of resistance^1^	Number of isolates	Resistance, %
No resistance	805	37.30
TilmicoTildiprTiamulinFlo	475	22.01
Tetra	142	6.58
TilmicoTildiprTiamulin	88	4.08
TetraTilmicoTildiprTiamulinFlo	81	3.75
Flo	63	2.92
TilmicoTildiprTulathGamithTiamulinFlo	60	2.78
TilmicoTildiprFlo	54	2.50
TilmicoTildiprGamithTiamulinFlo	50	2.32
TilmicoTildiprGamithFlo	27	1.25
TylosinTilmicoTildiprTulathGamithTiamulinFlo	27	1.25
TetraTilmicoTildiprFlo	23	1.07
TilmicoTildipr	22	1.02
TetraTilmicoTildiprTiamulin	19	0.88
TildiprTiamulinFlo	17	0.79
TilmicoTildiprTulathTiamulinFlo	16	0.74
TildiprTulathGamithTiamulinFlo	1	0.05
TilmicoFlo	1	0.05
Other combinations	Less than 1%	

**Note:**

1 Tilmico, Tilmicosin; Tildipr, Tildipirosin, Tiamulin; Flo, Florfenicol; Tetra, Tetracycline; Gamith, Gamithromycin.

## Discussion

### AMR in *E. coli* isolates

*Escherichia coli* isolates from this study were widely resistant to amphenicols (florfenicol) and sulphadimethoxine. However, the *E. coli* strains had low resistance to ampicillin, cephalosporins (ceftiofur), and aminoglycosides (gentamicin and neomycin). Interestingly, 83.31% of our *E. coli* isolates and 46.54% of ES isolates were resistant to florfenicol, a drug that is not currently used in adult dairy cattle. Similarly, Sawant et al. (2007) reported that 78% of fecal *E. coli* isolates from healthy lactating cattle in Pennsylvania dairy herds were resistant to florfenicol. Out of 3,334 *E. coli* isolates that were obtained from bovine fecal and gastrointestinal tract samples obtained from dairy cattle in New York and other Northeastern states between 2004 and 2011, 44.9% of isolates showed resistance to florfenicol. Florfenicol resistance was also reported in *E. coli* isolates obtained from beef cattle (Carson et al., 2008; Vidovic & Korber, 2006). Florfenicol is an antibiotic approved in the US since 1996 for veterinary use in beef and non-lactating dairy cattle for treatment of bovine respiratory disease (BRD). This drug is not labeled for use in female dairy cattle 20 months of age or older, or in calves to be processed for veal. The high resistance prevalence to florfenicol in fecal *E. coli* detected in the current study agreed with findings from other studies that reported increasing florfenicol resistance among bacteria of animal respiratory tract origin (Coetzee et al., 2019; Klima et al., 2011) and gut origin, including *Enterobacterales* (Ying et al., 2019). Resistance to florfenicol in bovine *E. coli* is attributed to the presence of the *floR* gene which is also capable of conferring cross-resistance to other antimicrobials (Cloeckaert et al., 2000). Multiple studies (Ying et al., 2019; White et al., 2000; CIPARS, 2002) showed that florfenicol resistance was genetically linked to tetracycline or sulfamethoxazole resistance and its persistence is likely related to co-selection with either tetracycline or sulfamethoxazole resistance genes. A previous study showed that the *cfr* (C) gene and MDR/virulence plasmid is present in *Campylobacter coli* isolated from the intestinal cecal content of cattle from the United States, where it confers resistance to several antimicrobials including florfenicol, clindamycin, and tiamulin (Zhao et al., 2019).

Macrolides, including tilmicosin, tulathromycin, tylosin, tildipirosin and gamithromycin, are antimicrobial agents approved for treatment of BRD in the United States (Evans, 2005). *E. coli* is reported to have intrinsic resistance to the macrolide’s erythromycin, clarithromycin, and azithromycin (CLSI, 2018b). It is theorized that macrolides are hydrophobic substances that may not penetrate the outer membrane of certain Gram-negative bacteria, including *E. coli* and *Salmonella* spp. (Vaara, 1992; Pyörälä et al., 2014). Such a property may explain the high proportion of resistance of *E. coli* isolates in our study to some of the macrolides, including tilmicosin (99.86%), tylosin (99.86%) and gamithromycin (74.04%). Under that hypothesis, our study found that the MDR prevalence in our *E. coli* isolates was 14.14% without including apparent macrolide resistance. However, further research is needed on the nature of any resistance given that other macrolides showed greater effectiveness. Specifically, our study *E. coli* isolates showed very high susceptibility to tulathromycin (97.84%) and high susceptibility to tildipirosin (79.5%). Assuming some form of susceptibility to pleuromutilin and macrolides, the earlier estimate of MDR would be 38.1% were these drugs included in calculation of MDR.

Similarly, Tyson et al. (2015) reported high susceptibility of *E. coli* to macrolides with less than 5% phenotypic resistance to azithromycin. In contrast, other studies considered resistance to macrolides as acquired resistance (Leclercq, 2002; Szmolka & Nagy, 2013; Kadlec et al., 2011; Cazera et al., 2020). More than 70 genes encoding acquired macrolide resistance are hosted by more than 60 different bacterial species (Van Hoek et al., 2011) including *E. coli*. A *cfr* gene encoding for an unusual rRNA methylase and conferring a multidrug resistance phenotype (including resistance to lincosamides, streptogramins A, phenicols, pleuromutilins, and oxazolidinones) has been detected in several bacterial species, including *Enterococci* and *E. coli* from food animals (Schwarz, Kehrenberg & Ojo, 2002; Liu et al., 2012; Zhang et al., 2014). The most common resistance mechanism against macrolide antimicrobials is a target site modification mediated by at least 36 different rRNA methylases (*erm* genes) (Palmieri et al., 2013). These genes have been detected in many food animal pathogens such as *Enterococcus, M. haemolytica* and *P. multocida* (Kadlec et al., 2011). *Erm* genes can be transferred horizontally because of the association with mobile genetic elements (Roberts, 2011). Future genetic analysis should be considered to verify the cause of commensal *E. coli* resistance to macrolides.

A high proportion *E. coli* isolates in our study demonstrated resistance to tiamulin (98.93%). However, tiamulin is a semi-synthetic derivative of pleuromutilin that is used exclusively in pigs and poultry and is not approved for use in cattle. The *cfr*-mediated resistance has also been detected in *E. coli* of bovine origin (Long et al., 2006; Wang et al., 2012). The latter further supports the explanation that *E. coli* resistance to pleuromutilins, as with some macrolides, could be conferred due to another mechanism that is not gene related. In contrast, resistance of *Enterococcus* spp. to pleuromutilins in our study could be conferred due to the spread of resistance genes and MDR plasmids. The *cfr* gene, which is responsible for tiamulin resistance, has been detected in bacteria from nasal swabs of pigs in China (Wang et al., 2012). Similarly, Liu et al. (2012) reported the occurrence of the *cfr* gene in *Enterococcus faecalis* isolated from bovine and pig feces in China.

Our results showed low prevalence of resistance of fecal *E. coli* from the adult dairy cows to antimicrobials commonly administered to adult dairy cows such as cephalosporins and penicillin. Resistance of *E. coli* to ampicillin in this study was much lower than reported in the NARMS report (2016–2017). The prevalence of *E. coli* resistance in the current study is consistent with findings from a study evaluating AMR of fecal *E. coli* collected from dairy cows in 21 states in the US at one sampling point (Lundin et al., 2008) where they found that 20% or fewer of *E. coli* isolates were resistant to tetracycline (20%), streptomycin (6.4%), ampicillin (4.3%), ceftiofur (2.1%) and gentamicin (0.2%). NARMS (2016) reported a similar level of *E. coli* resistance to gentamicin and trimethoprim-sulfamethoxazole as compared to our study.

The most widely used antimicrobial drug class for dry-cow treatment and prevention of mastitis on the study dairies was cephalosporins (ceftiofur hydrochloride, cephapirin sodium). However, only 35 of the 2,169 fecal *E. coli* isolates (1.93%) were resistant to ceftiofur which suggested that these drugs are still effective in dairy cows. In the NAHMS Dairy 2014 study (USDA, 2018), cephalosporins were used as the primary antimicrobials to treat mastitis on 63.2% of operations; first-generation cephalosporins were used by 29.8% of operations and third generation cephalosporins by 33.4%. Low levels of *E. coli* resistance (<5%) to ceftiofur, aminoglycosides, trimethoprim-sulfamethoxazole, and fluoroquinolones were reported in previous studies on fecal samples of dairy cows (Carson et al., 2008; Boireau et al., 2018; Aasmäe et al., 2019) and feedlot cattle (USDA, 2007; Gow et al., 2008; Benedict et al., 2013; Waldner et al., 2019).

Our study showed low proportions of *E. coli* resistance to tetracycline (16.83%) in comparison to fecal *E. coli* resistance to tetracycline as reported in the NARMS, 2016 report and other dairy studies of AMR in fecal commensal bacteria (Cummings, Aprea & Altier, 2014; Boireau et al., 2018; Aasmäe et al., 2019). Such a difference could be due to spatiotemporal changes between the studies, specifically due to implementation of SB 27 in CA prior to initiation of the current study. However, 91.3% of bovine *E. coli* isolates obtained from clinical samples submitted to Cornell University’s Animal Health Diagnostic Center between 2004 and 2011 were resistant to oxytetracycline (Cummings, Aprea & Altier, 2014). The higher AMR of fecal *E. coli* to tetracyclines detected in the Cornell study compared to our study could be attributed to the fact that their samples were collected predominantly from calves with clinical diseases.

Although, enrofloxacin is not approved for use in adult lactating dairy cattle, we did observe resistance to enrofloxacin. Resistance to enrofloxacin in the current study was estimated at 3.31% which is similar to the enrofloxacin resistance rate (2.7%) reported by a study conducted on 3,373 bovine fecal *E. coli* isolates collected from dairy cattle in the Northeastern US from 2004 to 2011 (Cummings, Aprea & Altier, 2014). It has been observed that quinolone-resistant *E. coli* are more common in calves than in older cattle (Duse et al., 2015). The fecal microbiota of calves may serve as a reservoir for quinolone-resistant *E. coli* on dairies, causing exposure of adult cattle to resistant *E. coli*. A recent study (Liu et al., 2019) showed that the dairy calves’ intestines served as a reservoir of up to 329 antimicrobial genes (ARGs) which confer resistance to 17 classes of antibiotics. Future research is needed to look at the possibility of transmission of resistant bacteria from calves to adult cows on dairies.

### Prevalence of *E. coli* resistance across seasons

Our study showed seasonal difference in AMR of *E. coli* isolates, with more resistance observed during the winter compared to the summer season. Specifically, the current study showed that *E. coli* isolates had a higher prevalence of resistance to ceftiofur, tetracycline, fluoroquinolones, aminoglycosides (except neomycin), sulphadimethoxine and trimethoprim-sulfamethoxazole in winter compared to the summer season. The seasonal difference in AMR could be attributed to weather changes with the temperate, rainy winter in CA providing a favorable environment for bacteria to proliferate and colonize cows. The rainy season in CA typically begins in late November or early December. Mastitis has been known to increase in wet conditions because of muddy paddocks and bedding, which increases hygiene and management challenges to dairy producers during these times. The study conducted in CA by Gonzalez et al. (1990) showed that coliform mastitis is more prevalent in the rainy late fall and winter months (from October to March). It is possible that a higher prevalence of mastitis in fall through winter may be associated with more AMD use and, hence, an increase in resistant bacterial proportions. The increased resistance could also be attributed to higher disease incidence such as metritis, lameness, and displaced abomasum in the fall and winter (Ribeiro et al., 2013) when more cows calve; antimicrobial drugs are indicated for use in treatment of these diseases. Seasonal difference in AMR was also observed in a study by Watson et al. (2012) that found significant increases in shedding of antimicrobial resistant *E. coli* in May and August in comparison to November. Karzis et al. (2019) showed seasonal variations in the prevalence of *Staphylococcus aureus* antibiotic resistance in South African dairy herds over an 11-year study period where a higher prevalence of antibiotic resistance of *S. aureus* to cephalosporins was observed during the rainy and muddy season in comparison to the dry season. Overall, seasonal variation in AMR could be caused by many factors, such as the seasonal variation of infectious diseases, differences in management practices, weather conditions, seasonal differences between geographical region, or differences in antibiotic prescription patterns, all of which influence the dynamic interaction between host and environment and can exert antibiotic selective pressure.

### Prevalence of *E. coli* resistance across CA regions

Differences in *E. coli* AMR were seen across CA regions, with lower AMR reported in NCA compared to NSJV and GSCA. The lowest resistance of *E. coli* to cephalosporins was observed in NCA compared to NSJV and GSCA, which is consistent with our results regarding antimicrobial usage, especially at dry-off, which showed lower usage on NCA study herds as compared to study herds from the remaining regions. The difference in the use of intramammary antibiotics (lactating and dry-cow therapies) in the different regions could have contributed to a difference in bacterial antibiotic resistance across the different study regions. Two out of three dairies in NCA did not use any antimicrobials for prevention and treatment of clinical mastitis, which was mostly treated by cephalosporins in other CA regions. Additionally, the regional differences in AMR seen in our study could be related to other factors, including differences in environmental conditions and management practices. Two out of three dairies in NCA housed their cows in either a pasture system or mixed pasture and freestall barns in comparison to complete freestall barns in other CA regions. These regional differences in management and environmental conditions could influence incidence of disease and drug use, thereby contributing to the observed differences in AMR between regions. The results of a survey conducted in CA (unpublished finding) showed that approximately 8, 40 and 62 cows/month were treated with antibiotics for mastitis in NCA, NSJV and GSCA, respectively. The low *E. coli* AMR in NCA could be attributed to the fact that the two dairies enrolled from this region did not use antibiotics to prevent or treat mastitis. Also, the differences in herd size among CA regions may have contributed to AMR differences. The average herd size in NCA (803 cows/herd) was much lower than the average herd sizes in NSJV and GSCA (1,125 and 2,279 cows/herd respectively). The percentage of diseases (mastitis, metritis, lameness) that require antibiotic treatment were much lower in small herd operations as compared to medium and large herd operations according to a USDA report (2018).

### Prevalence of *E. coli* AMR across sampling points

Our results showed higher resistance of fecal *E. coli* around peak milk production period, at approximately 60 DIM. The higher resistance at 60 DIM could be a result of increased antimicrobial drug use during the post-calving due to the cow’s increased susceptibility to diseases, as reported by previous studies (Goff & Horst, 1997). Studies on adult cattle indicate that the shedding of antimicrobial-resistant *E. coli* increases around parturition (Watson et al., 2012; Callens et al., 2015). The high proportion of resistant *E. coli* observed postpartum could be attributed to the high risk of diseases within the first 21 days of calving and associated high probability of exposure to AMD, which co-select for AMR (Singer, Patterson & Wallace, 2008). Metritis caused by bacterial infection affects between 21% and 40% of dairy cattle and occurs within 21 days after parturition (Sheldon et al., 2006). Antimicrobial drugs commonly used for the treatment of metritis include penicillin, third generation cephalosporins, or a combination of ampicillin with oxytetracycline (Nak et al., 2011). According to the USDA (2018), mastitis affects about 24% of cows and most cows affected with mastitis (85.6%) were treated with antimicrobials. Pol & Ruegg (2007) and Saini et al. (2012) reported that the higher susceptibility of dairy cows to clinical mastitis involving coliform bacteria has been observed at calving and early lactation. A study of Watson et al. (2012) showed that cows shed more antimicrobial resistant *E. coli* post-calving compared to pre-calving. For postpartum cows, poor hygiene and group-pen calving were associated with increased odds of shedding resistant *E. coli* (Duse et al., 2015).

### Multidrug resistance in *E. coli* isolates

In the current study, approximately 14.14% of *E. coli* isolates were resistant to three or more antimicrobial drug classes, while 13.18% were pan-susceptible. The most common resistance phenotypic pattern observed for *E. coli* isolates in our study was resistance against florfenicol (45.8%), florfenicol and sulphadimethoxine (16.2%), and tetracycline-florfenicol-sulphadimethoxine (6.82%). Sawant et al. (2007) observed that the major multidrug resistance pattern in fecal *E. coli* isolated from dairy cattle in Pennsylvania was florfenicol and tetracycline (35.87%), and ampicillin-florfenicol-tetracycline (13.9%). Furthermore, Cummings, Aprea & Altier (2014) found that the most common resistance patterns were ampicillin-ceftiofur-trimethoprim-sulfamethoxazole (18.0%), ampicillin-trimethoprim-sulfamethoxazole (16.9%), ampicillin (13.6%) and pan-susceptible (12.8%). In contrast, Aasmäe et al. (2019) estimated that 40% of *E. coli* isolates from fecal samples of adult dairy cattle in Estonia were MDR. In our study, the most common resistance pattern observed among *E. coli* isolates included florfenicol. This result agrees with results obtained by a study conducted in Pennsylvania (Sawant et al., 2007) that reported that 78% of fecal *E. coli* isolates from healthy, lactating cattle were resistant to florfenicol. The observed multidrug resistance pattern in *E. coli* isolates obtained in our study could be attributed to the presence of multidrug resistance *cfr* gene in bovine *E. coli* isolates; however, further genetic analyses are needed to confirm this hypothesis. In *staphylococci*, the *cfr* gene mediates multidrug resistance to phenicols, lincosamides, oxazolidinones, pleuromutilins, streptogramin A, and the 16-membered macrolides spiramycin and josamycin (Long et al., 2006).

### AMR in *Enterococcus* spp./*Streptococcus* spp.

Recent taxonomical revision resulted in the new genus *Enterococcus* with several new species which were otherwise classified as *Streptococcus* (Pinto et al., 1999). As a result, approximately 44% of the study isolates were indeed *Enterococcus* spp., based on 16S partial gene sequencing and MALDI-ToF testing of a stratified random sample. The remaining 56% isolates were confirmed as *Streptococcus* spp. which may be explained by the similarity between *Enterococcus* spp. and *Streptococcus* spp. in colony morphology on the specific ChromoSelect media. The CLSI ampicillin and florfenicol breakpoints recommended for both *Enterococcus* and *Streptococcus* were identical, hence the AMR status determination for the ES isolates for these drugs were similar. For the remaining drugs (penicillin, tetracycline, tiamulin, gamithromycin, tilmicosin, tildipirosin, tulathromycin and tylosin) the breakpoints were consistently lower for *Streptococcus* spp. compared to *Enterococcus* spp. As a result, our study estimates could have potentially underestimated the *Streptococcus* spp. AMR to the aforementioned drugs.

Antimicrobial resistance in ES was monitored to understand resistance to antibiotics active against Gram-positive bacteria. The ES isolated from adult cows were highly resistant to macrolides (tildipirosin and tilmicosin), phenicols (florfenicol) and pleuromutilins (tiamulin). Macrolides belong to the category IV of critically important antimicrobial drugs for human medicine (USDA, 2018) and have been the first-line treatment against BRD in calves; they are also used to treat infections in humans (Zaheer et al., 2013). In the US, about one-third of preweaned heifers are treated for respiratory disease; and florfenicol was the primary antimicrobial for treating respiratory disease (USDA, 2018). High levels of resistance to macrolides and florfenicol have also been reported for enterococci isolated from dairy cows in several studies (Liu et al., 2012; Iweriebor, Obi & Okoh, 2016; Li et al., 2018).

Our results showed that ES isolated from the feces of dairy cows have a low level of resistance (<1%) to ampicillin and penicillin, which is consistent with results obtained from a study conducted on fecal samples collected from dairy cattle in 17 US states (Jackson et al., 2010). However, as shown in Table 5, the ES resistance to penicillin (0.18%) in our study was slightly higher than that reported by NARMS from 2013 to 2017. Penicillin resistance in *Enterococcus faecalis* has not been identified in isolates across all NARMS reports from 2013 to 2017. Penicillin resistance in *Enterococcus faecium* ranged from 0.00 to 3.92% across all NARMS reports. According to the USDA (2018), penicillin was the primary antimicrobial used for treatment of umbilical infections in preweaned heifers on 18.7% of US dairy operations. In addition, 10% of dairy operations used penicillin for the treatment of mastitis, reproductive disease, and respiratory disease in adult cows (USDA, 2018). The most widely used antimicrobial combinations for dry-cow treatment and prevention of mastitis were either penicillin/novobiocin or penicillin G/dihydrostreptomycin, which suggests that penicillins still appear to be clinically effective against ES (Jackson et al., 2010; De Oliveira et al., 2000). In cattle, ES have been associated with bovine mastitis in dairy cattle (Madsen et al., 1974; Rogers, Zeman & Erickson, 1992). Enterococci associated with mastitis are considered environmental pathogens as they are transmitted between the environment and the animal, rather than from animal to animal (Rossitto et al., 2002). On dairy farms, mastitis is one of the leading causes of antimicrobial use (Mitchell et al., 1998; USDA, 2005, 2008). Additionally, our results showed that the ES resistance to tetracycline and tylosin fall in the range reported by NARMS (2016). Similarly, the resistance of ES to tetracycline (24.5%) and tylosin (1.1%) was also reported in enterococci isolated from fecal samples collected in 2007 from US dairy cattle (Jackson et al., 2010).

### Prevalence of *Enterococcus* spp./Streptococcus spp. resistance across seasons

Possible reasons for the high prevalence of AMR of ES during the summer season observed in our study are the influence of climate and seasonal temperature differences and management practices. Sinton et al. (2007) looked at the seasonality of bovine fecal indicator organisms including enterococci on New Zealand farms over the course of a year and found that, although Enterococci counts in feces of dairy cows were variable, the highest counts occurred in spring and summer. Previous research conducted by Edrington et al. (2009) suggested that exposure of lactating cows to heat stress in summer is associated with energy loss which may result in heat-stressed cows shedding more resistant bacteria. The same study suggested that the increased use of sprinklers during summer produces moist conditions and establish a suitable environment for bacterial survival. Similarly, Edrington et al. (2006) showed that the incidence of *Enterococcus* spp. increased as pen soil moisture levels increased in cattle pens. Seasonal differences for *Enterococcus faecalis* and *Enterococcus faecium* were also observed for bovine raw milk samples, with the prevalence of *Enterococcus faecalis* increasing in summer and that of *Enterococcus faecium* increasing in autumn. *Enterococcus faecalis* remained the most prevalent species isolated in every season, including spring, which is the peak milking season (McAuley et al., 2015).

### Prevalence of *Enterococcus* spp./*Streptococcus* spp. resistance across regions

Our findings showed a significant difference in ES MDR across CA regions. Less resistance among enterococci isolates from NCA was observed as compared with those from NSJV and GSCA. The difference among regions could be attributed to differences in management practices, environmental conditions, herd size and the use of AMD in dry cows for treatment and prevention of mastitis. A survey conducted in Australia (McAuley et al., 2015) found that the *Enterococcal* microflora varied between dairy regions and over time, due to differences in climate and seasonal temperature differences, water supply, and the specific microflora on farms. The greater resistance to penicillins (ampicillin and penicillin) observed in enterococci isolates obtained from NCA, in comparison to those obtained from NSJV or GSCA, may be merely attributed to isolating *Enterococcus faecium* more frequently in NCA than elsewhere. *Enterococcus faecium* is reportedly more resistant to penicillin and ampicillin than other enterococci (Hollenbeck & Rice, 2012); however, the current study did not speciate the *Enterococcus* spp. isolates.

### Multidrug resistance in *Enterococcus* spp.*/Streptococcus* spp. across sampling points

Similar to our *E. coli* findings, our results showed higher resistance of fecal ES for most AMD at approximately 60 DIM compared to the remaining sampling points. According to the USDA (2018), AMD are commonly used for treatment of mastitis and metritis in dairy cows. Goto et al. (2019) reported that the percentage of cows with diseases within 60 days post-calving was 42%. The major categories of diseases were perinatal (34%), udder (18%) and metabolic (17%) diseases (Goto et al., 2019). Mastitis was the most common disease of dairy cows and the most common antibiotic treatment indication (Pol & Ruegg, 2007; Saini et al., 2012). Enterococci are one of the environmental pathogens associated with mastitis (Rossitto et al., 2002). The high proportion of resistant ES observed postpartum could be attributed to the high risk of diseases after calving and associated high probability of exposure to AMD (Singer, Patterson & Wallace, 2008).

### Multidrug resistance in *Enterococcus* spp.*/Streptococcus* spp. isolates

Multidrug resistance was also observed among ES isolates (38.64%). The majority of MDR ES isolates were resistant to three antimicrobials, but resistance to 5 antimicrobials was also observed for one isolate. Most importantly, about 37.31% of ES isolates were pan-susceptible to the five antimicrobial classes tested in our study, which agrees with reports from other countries (Aasmäe et al., 2019; DANMAP, 2015). Also, multidrug resistance in *Enterococcus* spp. from fecal samples of dairy cattle has been previously reported by studies conducted in the US (Jackson et al., 2010; Edrington et al., 2009).

### Study limitations

The current findings quantify the AMR of indicator organisms on dairies and their clustering by drug to which resistance was detected, and production stage of their source cows at the time of sample collection. And while we present here descriptive epidemiology on AMR by various management styles covering a wide geographic area, models for the association between treatment and AMR will be the subject of several future publications with the goal of devising control and prevention measures for AMR. Specifically, statistical models to investigate the association between antimicrobial drug use and AMR, and the strength of the association if any, adjusted for confounding factors and investigating any effect modifiers of this association will be explored.

Bacterial isolates of the current study were identified using selective media which rely on the morphology and biochemical properties of microorganisms. Funding limitations and the large number of isolates tested in the current study precluded us form testing more than two isolates per fecal sample and speciation of all 4,329 isolates beyond the confirmation offered by using specific media. However, a stratified random sample of isolates was speciated using MALDI-ToF and 16S rRNA partial gene sequencing. Despite the low accuracy (44%) of the *Enterococcus* spp. specific medium, the accuracy of the *E. coli* specific medium was excellent (98%). Furthermore, genotyping of the isolates’ resistance will be the subject of a different report and key to understanding the role of antimicrobial drug use, co-selection, horizontal gene transfer, and the environment as a repository of AMR genes. Such information is paramount to understanding the role of selection pressure, bacterial factors and the environment on resistance and the magnitude of their contribution, if any, to AMR on dairies.

## Conclusions

The current study detected very low resistance proportions to drugs commonly administered to adult dairy cows, such as cephalosporins and penicillins. We detected higher rates of AMR to drugs not approved for use in lactating cattle, such as florfenicol, that may be administered to calves rather than to adult cows. Further research is needed understand the determinants and mechanisms with which AMR against florfenicol occurs in fecal commensals from adult cows: specifically, the role of co-selection for AMR genes that confer resistance to drugs from different drug classes. To the best of our knowledge, the current study is the first across the US that has generated such a large repository of isolates from a diverse population of dairy cows representing a wide variety of management styles and across different regions and seasons. Overall, a higher prevalence of multidrug resistant isolates was observed in NSJV and GSCA in comparison to NCA. The information from this study provides a baseline measure for AMR in CA dairies that can be used for future references.

## Supplemental Information

10.7717/peerj.11108/supp-1 Supplemental Information 1 Interpretive categories and MIC breakpoints used to determine resistance for *Escherichia coli* isolates obtained from fecal samples of dairy cattle (mg/mL). ^1^Analysis cut-off concentration used for determining resistance. Isolates falling within the intermediate range were considered resistant ^2^ Because there is no interpretive criterion for neomycin, we used the breakpoint for gentamicin as both antibiotics are aminoglycosides.
^3^Because no spectinomycin MIC breakpoint was available for bovine *E. coli* by CLSI guidelines, the breakpoints for gram-negative bovine respiratory agents were used as a guideline.

10.7717/peerj.11108/supp-2 Supplemental Information 2 Interpretive Categories and MIC breakpoints used to determine resistance for *Enterococcus* spp./ *Streptococcus* spp. isolates from fecal samples of cattle (μg/mL). ^1^Analysis cut-off concentration used for determining resistance. Isolates falling within the intermediate range were considered resistant

10.7717/peerj.11108/supp-3 Supplemental Information 3 Proportion of resistance in *E. coli* isolated from fecal samples of California dairy cows in different regions of CA over winter cohort.

10.7717/peerj.11108/supp-4 Supplemental Information 4 Proportion of resistance in *E. coli* isolated from fecal samples of California dairy cows in different regions of CA over summer cohort.

10.7717/peerj.11108/supp-5 Supplemental Information 5 Proportion of resistance in *Enterococcus spp./ Streptococcus spp*. isolated from fecal samples of California dairy cows in different regions of CA over winter cohort. Due to difference in breakpoints for these drugs between Enterococcus spp. and Streptococcus spp., the estimates should be interpreted with caution for Streptococcus spp. due to potential overestimation of the susceptibility.

10.7717/peerj.11108/supp-6 Supplemental Information 6 Proportion of resistance in Enterococcus spp./ Streptococcus spp. isolated from fecal samples of California dairy cows in different regions of CA over summer cohort. Due to difference in breakpoints for these drugs between *Enterococcus* spp. and *Streptococcus* spp. , the estimates should be interpreted with caution for *Streptococcus* spp. due to potential overestimation of the susceptibility.

10.7717/peerj.11108/supp-7 Supplemental Information 7 Proportion of resistance in *Escherichia coli* isolated from fecal samples of California dairy cows during different sampling points over two cohorts from 2018 to 2019.

10.7717/peerj.11108/supp-8 Supplemental Information 8 Proportion of resistance in *Escherichia coli* isolated from fecal samples of dairy cows during different sampling points over winter cohort.

10.7717/peerj.11108/supp-9 Supplemental Information 9 Proportion of resistance in *Escherichia coli* isolated from fecal samples of California dairy cows over sampling points during summer season.

10.7717/peerj.11108/supp-10 Supplemental Information 10 Proportion of resistance in *Enterococcus* spp./ *Streptococcus* spp. isolates originated from fecal samples of California dairy cows over sampling points over two cohorts from 2018 to 2019. Due to difference in breakpoints for these drugs between *Enterococcus* spp. and *Streptococcus* spp. , the estimates should be interpreted with caution for *Streptococcus* spp. due to potential overestimation of the susceptibility.

10.7717/peerj.11108/supp-11 Supplemental Information 11 Proportion of resistance in *Enterococcus* spp./ *Streptococcus* spp. isolated from fecal samples of California dairy cows during different sampling points over winter cohort 2018-2019. Due to difference in breakpoints for these drugs between *Enterococcus* spp. and *Streptococcus* spp., the estimates should be interpreted with caution for *Streptococcus* spp. due to potential overestimation of the susceptibility.

10.7717/peerj.11108/supp-12 Supplemental Information 12 Proportion of resistance in Enterococcus *spp./* Streptococcus *spp*. isolated from fecal samples of California dairy cows over sampling points during summer cohort. Due to difference in breakpoints for these drugs between *Enterococcus* spp. and *Streptococcus* spp., the estimates should be interpreted with caution for *Streptococcus* spp. due to potential overestimation of the susceptibility.

## Additional Information and Declarations

### Data Availability

The following information was supplied regarding data availability: This study was sponsored by the California Department of Food and Agriculture and is subject to California Food and Agriculture Code (FAC) Sections 14400 to 14408. FAC section 14407 requires that data collected be held confidential to prevent individual identification of a farm or business; as such, raw data from this study is not able to be shared.
