# Supplementary material for: Epidemiology of antimicrobial resistance (AMR) on California dairies: descriptive and cluster analyses of AMR phenotype of fecal commensal bacteria isolated from adult cows
Source: PeerJ. 2021 Apr 20;9:e11108. doi: 10.7717/peerj.11108 (PMC8063881; doi:10.7717/peerj.11108)
Supplement: Supplemental Information 1 — 1Analysis cut-off concentration used for determining resistance. Isolates falling within the intermediate range were considered resistant 2 Because there is no interpretive criterion for neomycin, we used the breakpoint for gentamicin as both antibiotics are aminoglycosides. 3Because no spectinomycin MIC breakpoint was available for bovine E. coli by CLSI guidelines, the breakpoints for gram-negative bovine respiratory agents were used as a guideline. [file peerj-09-11108-s001.docx]

ST1. Interpretive categories and MIC breakpoints used to determine resistance for *Escherichia coli* isolates obtained from fecal samples of dairy cattle (mg/mL)

| Antimicrobial Class | Antimicrobial Drugs | Susceptible | Intermediate | Resistant | Analysis cut-off^1^ | Source | References |
| --- | --- | --- | --- | --- | --- | --- | --- |
| Penicillins | Ampicillin | ≤8 | 16 | >16 | ≥16 | CLSI VET 08 (2018); CLSI M100(2017)-Table 2A-1 | Waldner et al., (2019); Carson et al., (2008); Gow et al., (2008) |
| Cephalosporins | Ceftiofur | ≤ 2 | 4 | ≥8 | ≥4 | CLSI VET 08 (2018) | Waldner et al., (2019); Carson et al., (2008) |
| Fluoroquinolones | Danofloxacin | ≤ 0.25 | 0.5 | ≥1 | ≥0.5 | CLSI VET08, (2018) | CLSI VET08, 4th edition (2018); Table 2G |
|  | Enrofloxacin | ≤ 0.25 | 0.5-1 | ≥2 | ≥0.5 | CLSI VET08, (2018); Table 2G | Watts &Sweeney (2010); Pereira et al., (2011) |
| Amphenicols | Florfenicol | ≤ 2 | 4 | ≥8 | ≥4 | CLSI VET08, (2018); Table 2G | CLSI VET08, 4th edition (2018); Table 2G |
| Aminoglycosides | Gentamicin | ≤4 | 8 | ≥16 | ≥8 | CLSI VET 08 (2018); CLSI M100-Table 2A-1, 2017 | Waldner et al., (2019); Benedict et al. (2013) Carson et al., (2008); Gow et al., (2008) |
|  | Neomycin^2^ | ≤4 | 8 | ≥16 | ≥8 | CLSI VET 08 (2018) | Pereira et al., (2011) |
|  | Spectinomycin^3^ | ≤32 | 64 | >64 | ≥64 | CLSI VET08 (2018); Table 2G. | Donaldson et al., (2006) |
| Sulfonamides | Sulphadimethoxine | <= 256 |  | >256 | >256 | CLSI M100-Table 2A-1, 2017 (sulfonamides). | Waldner et al., (2019; Carson et al., (2008) |
| Tetracyclines | Tetracycline | ≤2 | 4 | >8 | ≥4 | CLSI VET08, (2018); Table 2G; CLSI M100-Table 2A-1, 2017. | Waldner et al., (2019); Carson et al., (2008); Gow et al., (2008) |
| Folate pathway antagonist | Trimethoprim-sulfamethoxazole | ≤ 2/38 | . | > 2/38 | > 2/38 | VET 08 (2018); CLSI M100-Table 2A-1, 2017. | Waldner et al., (2019); Carson et al., (2008); Gow et al., (2008) |

References

Benedict, K.M., S.P. Gow, S. Checkley, C.W. Booker, T.A. McAllister, and P.S. Morley. 2013. Methodological comparisons for antimicrobial resistance surveillance in feedlot cattle. BMC Vet Res 9:216. doi:10.1186/1746-6148-9-216.

Carson, C.A., R. Reid-Smith, R.J. Irwin, W.S. Martin, and S.A. McEwen. 2008. Antimicrobial resistance in generic fecal Escherichia coli from 29 beef farms in Ontario 10. Can J Vet Res. 72(2): 119–128.

CLSI (Clinical and Laboratory Standards Institute). 2017. Performance standards for antimicrobial susceptibility testing. 27th ed. CLSI supplement M100. Wayne, PA: Clinical and Laboratory Standards Institute.

CLSI (Clinical and Laboratory Standards Institute). 2018. Performance standards for antimicrobial disk and dilution susceptibility tests for bacteria isolated from animals. 4^th^ ed. CLSI supplement VET08. Wayne, PA. Clinical and Laboratory Standards Institute.

Donaldson, S.C., B.A. Straley, N.V. Hegde, A.A. Sawant, C. DebRoy, and B.M. Jayarao. 2006. Molecular epidemiology of ceftiofur-resistant *Escherichia coli* isolates from dairy calves. Applied and Environmental Microbiology. 72:3940–3948. doi:10.1128/AEM.02770-05.

Gow, S.P., C.L. Waldner, A. Rajic, M.E. McFall, and R. Reid-Smith. 2008. Prevalence of antimicrobial resistance in fecal generic *Escherichia coli* isolated in western Canadian. Part II — Cows and cow-calf pairs. Can J Vet Res. 2008 Mar; 72(2): 91–100.

Pereira, R.V.V., T.M.A. Santos, M.L. Bicalho, L.S. Caixeta, V.S. Machado, and R.C. Bicalho. 2011. Antimicrobial resistance and prevalence of virulence factor genes in fecal *Escherichia coli* of Holstein calves fed milk with and without antimicrobials. Journal of Dairy Science 94:4556–4565. doi:10.3168/jds.2011-4337.

Waldner, C.L., S. Gow, S. Parker, and J.R. Campbell. 2019. Antimicrobial resistance in fecal Escherichia coli and Campylobacter spp. from beef cows in western Canada and associations with herd attributes and antimicrobial use 10. Can J Vet Res. 2019 Apr; 83(2): 80–89.
