# Supplementary material for: Epidemiology of antimicrobial resistance (AMR) on California dairies: descriptive and cluster analyses of AMR phenotype of fecal commensal bacteria isolated from adult cows
Source: PeerJ. 2021 Apr 20;9:e11108. doi: 10.7717/peerj.11108 (PMC8063881; doi:10.7717/peerj.11108)
Supplement: Supplemental Information 2 — 1Analysis cut-off concentration used for determining resistance. Isolates falling within the intermediate range were considered resistant [file peerj-09-11108-s002.docx]

Table S2. Interpretive Categories and MIC breakpoints used to determine resistance for *Enterococcus* spp./ *Streptococcus* spp. isolates from fecal samples of cattle (μg/mL)

| Antimicrobial Drugs | Susceptible | Intermediate | Resistant | Analysis cut-off^1^ | Sources | Citation |
| --- | --- | --- | --- | --- | --- | --- |
| Ampicillin | ≤8 | - | ≥16 | ≥16 | CLSI VET08, (2018) Human value; Table 2E | Barlow et al., (2017) |
| Florfenicol | - | 4 | ≥8 | ≥4 | VET08, (2018); Table1 | Liu et al., (2013); Liu et al., (2018) |
| Penicillin | ≤8 | - | >8 | >8 | CLSI VET08, (2018) Human value; CLSI M100-Table 2D | Liu et al., (2018); Barlow et al., (2017) |
| Tetracycline | ≤2 | 4 | >8 | ≥4 | CLSI VET08, (2018) Human value; CLSI M100-Table 2D | Barlow et al., (2017); Liu et al., (2018) |
| Tiamulin | - | - | ≥32 | ≥32 | Adopted from *E. coli* (Table 2) | Schwarz et al., (2016); Hollenbeck et al., (2012) |
| Gamithromycin |  |  | >8 | ≥8 | Adopted from *E. coli* (Table 2) | Tian et al., (2019); Hollenbeck et al., (2012); Portillo et al., (2000) |
| Tildipirosin | ≤4 | 8 | >16 | ≥8 | Adopted from *E. coli* (Table 2) | Liu et al., (2018) |
| Tilmicosin | ≤8 | 16 | >16 | ≥16 | Adopted from *E. coli* (Table 2) | Liu et al., (2018) |
| Tulathromycin | ≤16 | 32 | >64 | ≥32 | Adopted from *E. coli* (Table 2) | Hollenbeck et al., (2012); Portillo et al., (2000) |
| Tylosin | ≤ 8 | 16 | >16 | ≥16 | Adopted from *E. coli* (Table 2) | Liu et al., (2018); Beukers et al., (2015); Hollenbeck et al., (2012); Portillo et al., (2000) |

References

Barlow, R.S., K.E. McMillan, L.L. Duffy, N. Fegan, D. Jordan, and G.E. Mellor. 2017. Antimicrobial resistance status of *Enterococcus* from Australian cattle populations at slaughter. PLOS ONE 12: e0177728. doi: 10.1371/journal.pone.0177728.

Beukers, A.G., R. Zaheer, S.R. Cook, K. Stanford, A.V. Chaves, M.P. Ward, and McAllister, T.A. 2015. Effect of in-feed administration and withdrawal of tylosin phosphate on antibiotic resistance in enterococci isolated from feedlot steers. Front. Microbiol. 6. doi:10.3389/fmicb.2015.00483.

Hollenbeck, B.L., and L.B. Rice. 2012. Intrinsic and acquired resistance mechanisms in *enterococcus*. Virulence 3:421–569. doi:10.4161/viru.21282.

Liu M, Kemper N, Volkmann N and Schulz J. 2018. Resistance of *Enterococcus* spp. in dust from farm animal houses: A retrospective study. Front. Microbiol. 9:3074. doi: 10.3389/fmicb.2018.03074

Liu, Y., Liu, K., Lai, J., Wu, C., Shen, J. and Wang, Y. 2013. Prevalence and antimicrobial resistance of *Enterococcus* species of food animal origin from Beijing and Shandong Province, China. Journal of Applied Microbiology 114, 555-563.

Portillo, A., F. Ruiz-Larrea, M. Zarazaga, A. Alonso, J.L. Martinez, and C. Torres. 2000. Macrolide resistance genes in *Enterococcus* spp. Antimicrobial Agents and Chemotherapy 44:967–971. doi:10.1128/AAC.44.4.967-971.2000.

Schwarz, S., J. Shen, K. Kadlec, Y. Wang, G. Brenner Michael, A.T. Feßler, and B. Vester. 2016. Lincosamides, streptogramins, phenicols, and pleuromutilins: Mode of action and mechanisms of resistance. Cold Spring Harb Perspect Med 6: a027037. doi:10.1101/cshperspect. a027037.

Tian, Y., H. Yu, and Z. Wang. 2019. Distribution of acquired antibiotic resistance genes among *Enterococcus* spp. isolated from a hospital in Baotou, China. BMC Res Notes 12:27. doi:10.1186/s13104-019-4064-z.
