# Supplementary material for: Epidemiology of antimicrobial resistance (AMR) on California dairies: descriptive and cluster analyses of AMR phenotype of fecal commensal bacteria isolated from adult cows
Source: PeerJ. 2021 Apr 20;9:e11108. doi: 10.7717/peerj.11108 (PMC8063881; doi:10.7717/peerj.11108)
Supplement: Supplemental Information 3 [file peerj-09-11108-s003.docx]

Table S3. Proportion of resistance in *E. coli* isolated from fecal samples of California dairy cows in different regions of CA over winter cohort

| Antimicrobial class | Antimicrobial drug | **Northern CA** | | **Northern San Joaquin Valley** | | **Greater Southern CA** | |
| --- | --- | --- | --- | --- | --- | --- | --- |
|  |  | **% ± SE** | **95% CI** | **%** | **95% CI** | **%** | **95% CI** |
| Penicillins | Ampicillin | 0.91 ± 0.52 | 0.29, 2.79 | 0.47 ± 0.47 | 0.06, 3.29 | 1.12 ± 0.45 | 0.50, 2.48 |
| Cephalosporins | Ceftiofur | 0.61 ± 0.42 | 0.15, 2.40 | 6.13 ± 1.65 | 3.58, 10.29 | 3.74 ± 0.82 | 2.42, 5.73 |
| Tetracyclines | Tetracycline | 17.32 ± 2.10 | 13.59, 21.81 | 21.69 ± 2.83 | 16.64 ± 27.77 | 24.34 ± 1.85 | 20.88, 28.17 |
| Fluoroquinolones | Enrofloxacin | 1.82 ± 0.73 | 0.82, 4.01 | 10.84 ± 2.14 | 7.31, 15.82 | 5.99 ± 1.02 | 4.26, 8.35 |
|  | Danofloxacin | 3.03 ± 0.95 | 1.64, 5.56 | 11.32 ± 2.18 | 7.69, 16.35 | 7.11 ± 1.11 | 5.21, 9.63 |
| Aminoglycosides | Gentamicin | 0.61 ± 0.42 | 0.51, 2.40 | 1.41 ± 0.81 | 0.45, 4.31 | 0.37 ± 0.26 | 0.09, 1.48 |
|  | Neomycin | 0.91 ± 0.52 | 0.29, 2.79 | 3.30 ± 1.23 | 1.57, 6.77 | 2.05 ± 0.61 | 1.13, 3.67 |
|  | Spectinomycin | 2.43 ± 0.85 | 1.21, 4.79 | 13.21 ± 2.33 | 1.21, 4.79 | 10.29 ± 1.31 | 7.98, 13.18 |
| Amphenicols | Florfenicol | 83.89 ± 2.02 | 79.50, 87.48 | 78.30 ± 2.83 | 72.22, 83.85 | 81.64 ± 1.67 | 78.12, 84.71 |
| Sulfonamides | Sulphadimethoxine | 25.22 ± 2.39 | 20.81, 30.21 | 39.62 ± 3.36 | 33.23, 46.38 | 44.56±2.15 | 40.39, 48.82 |
| Folate pathway antagonist | Trimethoprim-sulfamethoxazole | 2.21 ± 0.79 | 1.01, 4.40 | 12.26 ± 2.25 | 8.47, 17.42 | 9.55 ± 1.27 | 7.32, 12.35 |
