# Supplementary material for: Epidemiology of antimicrobial resistance (AMR) on California dairies: descriptive and cluster analyses of AMR phenotype of fecal commensal bacteria isolated from adult cows
Source: PeerJ. 2021 Apr 20;9:e11108. doi: 10.7717/peerj.11108 (PMC8063881; doi:10.7717/peerj.11108)
Supplement: Supplemental Information 4 [file peerj-09-11108-s004.docx]

Table S4. Proportion of resistance in *E. coli* isolated from fecal samples of California dairy cows in different regions of CA over summer cohort.

| Antimicrobial class | Antimicrobial drug | **Northern CA** | | **Northern San Joaquin Valley** | | **Greater Southern CA** | |
| --- | --- | --- | --- | --- | --- | --- | --- |
|  |  | % ± SE | 95% CI | % | 95% CI | % | 95% CI |
| Penicillins | Ampicillin | 0.31 ± 0.31 | 0.04, 2.16 | 2.38 ± 1.05 | 0.99, 5.61 | 1.25 ± 0.47 | 0.59, 2.61 |
| Cephalosporins | Ceftiofur | 0.31 ± 0.31 | 0.04, 2.16 | 0.47 ± 0.47^a^ | 0.06, 3.32 | 0.89 ± 0.39 | 0.37, 2.13 |
| Tetracyclines | Tetracycline | 11.10 ± 1.74 | 8.10, 14.98 | 14.76 ± 2.45 | 10.56, 20.24 | 11.62 ± 1.35 | 9.22, 14.56 |
| Fluoroquinolones | Enrofloxacin | 0.31 ± 0.31 | 0.04, 2.20 | 1.42 ± 0.82 | 0.45, 4.35 | 1.25 ± 0.47 | 0.59, 2.61 |
|  | Danofloxacin | 0.31 ± 0.31 | 0.04, 2.16 | 0.95 ± 0.67 | 0.23, 3.74 | 2.14 ± 0.61 | 1.22, 3.74 |
| Aminoglycosides | Gentamicin | 0.00 ± 0.00 | . | 0.00 ± 0.00 | 0 | 0.00 ± 0.00 | . |
|  | Neomycin | 0.61 ± 0.43 | 0.15, 2.43 | 1.42 ± 0.82 | 0.45, 4.35 | 1.61 ± 0.53 | 0.83, 3.10 |
|  | Spectinomycin | 2.46 ± 0.86 | 1.23, 4.85 | 0.00 ± 0.00 | 0 | 1.96 ± 0.58 | 1.10, 3.52 |
| Amphenicols | Florfenicol | 84.31 ± 2.02 | 79.92, 87.87 | 86.66 ± 2.35 | 81.34, 90.64 | 84.61 ± 1.52 | 81.37, 87.37 |
| Sulfonamides | Sulphadimethoxine | 27.38 ± 2.47 | 22.79, 32.50 | 27.14 ± 3.10 | 21.54, 33.57 | 27.37 ± 1.88 | 23.82, 31.22 |
| Folate pathway antagonist | Trimethoprim-sulfamethoxazole | 0.92 ± 0.53 | 0.29, 2.84 | 0.95 ± 0.67 | 0.23, 3.74 | 1.43 ± 0.50 | 0.72, 2.83 |
