# Supplementary material for: Epidemiology of antimicrobial resistance (AMR) on California dairies: descriptive and cluster analyses of AMR phenotype of fecal commensal bacteria isolated from adult cows
Source: PeerJ. 2021 Apr 20;9:e11108. doi: 10.7717/peerj.11108 (PMC8063881; doi:10.7717/peerj.11108)
Supplement: Supplemental Information 5 — Due to difference in breakpoints for these drugs between Enterococcus spp. and Streptococcus spp., the estimates should be interpreted with caution for Streptococcus spp. due to potential overestimation of the susceptibility. [file peerj-09-11108-s005.docx]

Table S5. Proportion of resistance in *Enterococcus* spp*./ Streptococcus* spp*.* isolated from fecal samples of California dairy cows in different regions of CA over winter cohort.

| Antimicrobial class | Antimicrobial drug | **Northern CA** | | **Northern San Joaquin Valley** | | **Greater Southern CA** | |
| --- | --- | --- | --- | --- | --- | --- | --- |
|  |  | % ± SE | 95% CI | % | 95% CI | % | 95% CI |
| Penicillins | Ampicillin | 0.89 ± 0.51 | 0.28, 2.75 | 0.00 ± 0.00 | 0 | 0.19 ± 0.19 | 0.02, 1.39 |
|  | Penicillin | 0.89 ± 0.51 | 0.28, 2.75 | 0.00 ± 0.00 | 0 | 0.00 ± 0.00 | 0 |
| Tetracyclines | Tetracycline | 17.36 ± 2.10 | 13.66, 21.82 | 6.13 ± 1.65 | 3.58, 10.29 | 11.10 ± 1.39 | 8.61, 14.11 |
| Pleuromutilins | Tiamulin | 16.46 ± 2.03 | 12.85, 20.85 | 47.64 ± 3.43 | 40.97, 54.39 | 41.30 ± 2.19 | 37.10, 45.65 |
| Macrolides | Gamithromycin | 5.38 ± 1.23 | 3.41, 8.40 | 18.39 ± 2.66 | 13.72, 24.21 | 10.47 ± 1.36 | 8.10, 13.46 |
|  | Tilmicosin | 23.95 ± 2.33 | 19.66, 28.83 | 50.23 ± 3.45 | 43.50, 56.96 | 41.69 ± 2.20 | 37.46, 46.05 |
|  | Tildipirosin | 23.95 ± 2.33 | 19.66, 28.83 | 53.30 ± 3.43 | 46.54, 59.94 | 44.86 ± 2.21 | 40.56, 49.23 |
|  | Tulathromycin | 0.59 ± 0.42 | 0.14, 2.37 | 14.15 ± 2.39 | 10.10, 19.54 | 6.71 ± 1.11 | 4.83, 9.26 |
|  | Tylosin | 1.49 ± 0.66 | 0.62, 3.54 | 8.96 ± 1.96 | 5.78, 13.64 | 2.96 ± 0.75 | 1.79, 4.86 |
| Amphenicols | Florfenicol | 21.55 ± 2.25 | 17.46, 26.30 | 53.77 ± 3.43 | 47.01, 60.40 | 44.26 ± 2.21 | 39.98, 48.63 |
