# Supplementary material for: Epidemiology of antimicrobial resistance (AMR) on California dairies: descriptive and cluster analyses of AMR phenotype of fecal commensal bacteria isolated from adult cows
Source: PeerJ. 2021 Apr 20;9:e11108. doi: 10.7717/peerj.11108 (PMC8063881; doi:10.7717/peerj.11108)
Supplement: Supplemental Information 6 — Due to difference in breakpoints for these drugs between Enterococcus spp. and Streptococcus spp. , the estimates should be interpreted with caution for Streptococcus spp. due to potential overestimation of the susceptibility. [file peerj-09-11108-s006.docx]

Table S6. Proportion of resistance in *Enterococcus* spp*./ Streptococcus* spp*.* isolated from fecal samples of California dairy cows in different regions of CA over summer cohort.

| Antimicrobial class | Antimicrobial drug | **Northern CA** | | **Northern San Joaquin Valley** | | **Greater Southern CA** | |
| --- | --- | --- | --- | --- | --- | --- | --- |
|  |  | % ± SE | 95% CI | % | 95% CI | % | 95% CI |
| Penicillins | Ampicillin | 0.30 ± 0.30 | 0.04,2.10 | 0.00 | . | 0.00 | . |
|  | Penicillin | 0.00 | . | 0.48 ± 0.48 | 0.06, 3.38 | 0.00 | . |
| Tetracyclines | Tetracycline | 19.76 ± 2.20 | 15.85, 24.35 | 19.42 ± 27.62 | 14.55, 25.41 | 16.96 ± 1.58 | 14.10, 20.31 |
| Pleuromutilins | Tiamulin | 37.46 ± 2.63 | 32.45, 42.75 | 51.94 ± 3.48 | 45.10, 58.71 | 56.25 ± 2.10 | 52.10, 60.31 |
| Macrolides | Gamithromycin | 12.38 ± 1.79 | 9.30, 16.35 | 13.59 ± 2.39 | 9.54, 19.00 | 12.32 ± 1.39 | 9.84, 15.32 |
|  | Tilmicosin | 52.21 ± 2.72 | 46.87, 57.49 | 57.76 ± 3.44 | 50.89 ± 4.35 | 60.71 ± 2.10 | 56.59, 64.68 |
|  | Tildipirosin | 52.51 ± 2.72 | 47.16, 57.78 | 62.62 ± 3.37 | 55.79, 68.98 | 63.51 ± 2.03 | 59.42, 67.40 |
|  | Tulathromycin | 8.25 ± 1.50 | 5.76, 11.71 | 12.13 ± 2.28 | 8.32, 17.36 | 8.21 ± 1.16 | 6.20, 10.79 |
|  | Tylosin | 2.94 ± 0.92 | 1.59, 5.40 | 3.39 ± 1.26 | 1.62, 6.97 | 2.32 ± 0.63 | 1.35, 3.96 |
| Amphenicols | Florfenicol | 44.54 ± 2.70 | 39.31, 49.88 | 51.94 ± 3.48 | 45.10, 58.71 | 60.00 ± 2.10 | 55.87, 63.98 |
