# Supplementary material for: Epidemiology of antimicrobial resistance (AMR) on California dairies: descriptive and cluster analyses of AMR phenotype of fecal commensal bacteria isolated from adult cows
Source: PeerJ. 2021 Apr 20;9:e11108. doi: 10.7717/peerj.11108 (PMC8063881; doi:10.7717/peerj.11108)
Supplement: Supplemental Information 7 [file peerj-09-11108-s007.docx]

Table S7. Proportion of resistance in *Escherichia coli* isolated from fecal samples of California dairy cows during different sampling points over two cohorts from 2018-2019.

| Antimicrobial class | Antimicrobial drug | Sampling points, days relative to calving | | | | |
| --- | --- | --- | --- | --- | --- | --- |
|  |  | Close-up | 30 | 60 | 90 | 120 |
| Penicillins | Ampicillin | 1.33 ± 0.54 | 1.12 ± 0.50 | 0.686 ± 0.39 | 0.96 ± 0.47 | 1.187 ± 0.53 |
| Cephalosporins | Ceftiofur | 1.11 ± 0.49 | 1.34 ± 0.547 | 3.66 ± 0.89 | 2.40 ± 0.75 | 1.187 ± 0.53 |
| Tetracyclines | Tetracycline | 22.00 ± 1.95 | 17.75 ± 1.81 | 13.27 ± 1.62 | 15.14 ± 1.76 | 15.67 ± 1.77 |
| Fluoroquinolones | Enrofloxacin | 3.77 ± 0.89 | 2.92 ± 0.79 | 6.86 ± 1.21 | 2.40 ± 0.75 | 0.47 ± 0.33 |
|  | Danofloxacin | 4.44 ± 0.97 | 3.37 ± 0.85 | 6.86 ± 1.21 | 3.60 ± 0.91 | 1.66 ± 0.62 |
| Aminoglycosides | Gentamicin | 0.22 ± 0.22 | 0.67 ± 0.38 | 0.457 ± 0.32 | 0.00 ± 0.00 | 0.24 ± 0.24 |
|  | Neomycin | 2.87 ± 0.78 | 1.348 ± 0.54 | 1.83 ± 0.64 | 0.96 ± 0.47 | 0.95 ± 0.47 |
|  | Spectinomycin | 4.22 ± 0.94 | 5.16 ± 1.05 | 8.92 ± 1.36 | 5.04 ± 1.07 | 1.90 ± 0.66 |
| Amphenicols | Florfenicol | 87.33 ± 1.56 | 84.04 ± 1.74 | 84.21 ± 1.74 | 80.28 ± 1.95 | 80.28 ± 1.94 |
| Sulfonamides | Sulphadimethoxine | 42.00 ± 2.33 | 38.65 ± 2.31 | 32.49 ± 2.24 | 27.40 ± 2.18 | 20.66 ± 1.97 |
| Folate pathway antagonist | Trimethoprim-sulfamethoxazole | 4.00 ± 0.92 | 4.269 ± 0.95 | 7.55 ± 1.26 | 5.769 ± 1.14 | 0.71 ± 0.41 |
