# Supplementary material for: Epidemiology of antimicrobial resistance (AMR) on California dairies: descriptive and cluster analyses of AMR phenotype of fecal commensal bacteria isolated from adult cows
Source: PeerJ. 2021 Apr 20;9:e11108. doi: 10.7717/peerj.11108 (PMC8063881; doi:10.7717/peerj.11108)
Supplement: Supplemental Information 8 [file peerj-09-11108-s008.docx]

Table S8. Proportion of resistance in *Escherichia coli* isolated from fecal samples of dairy cows during different sampling points over winter cohort.

| Antimicrobial class | Antimicrobial drug | Sampling points, days relative to calving | | | | |
| --- | --- | --- | --- | --- | --- | --- |
|  |  | Close-up | 30 | 60 | 90 | 120 |
| Penicillins | Ampicillin | 1.37 ± 0.79 | 0.92 ± 0.65 | 0.45 ± 0.45 | 0.94 ± 0.66 | 0.95 ± 0.67 |
| Cephalosporins | Ceftiofur | 0.91 ± 0.64 | 2.31 ± 1.02 | 7.27 ± 1.75^a^ | 4.24 ± 1.38 | 1.43 ± 0.82 |
| Tetracyclines | Tetracycline | 32.56 ± 3.18 | 25.92 ± 2.98 | 17.72 ± 2.58 | 15.56 ± 2.49 | 16.26 ± 2.55 |
| Fluoroquinolones | Enrofloxacin | 3.66 ± 1.27 | 6.01 ± 1.62 | 13.63 ± 2.31 | 4.71 ± 1.45 | 0.00 ± 0.00 |
|  | Danofloxacin | 4.58 ± 1.42 | 6.01 ± 1.62 | 13.18 ± 2.28 | 6.60 ± 1.70 | 2.87 ± 1.15 |
| Aminoglycosides | Gentamicin | 0.45 ± 0.45 | 1.38 ± 0.79 | 0.90 ± 0.64 | 0.00 ± 0.00 | 0.47 ± 0.47 |
|  | Neomycin | 3.63 ± 1.26 | 1.85 ± 0.91 | 2.27 ± 1.00 | 0.47 ± 0.47 | 1.43 ± 0.82 |
|  | Spectinomycin | 5.50 ± 1.54 | 8.79 ± 1.93 | 16.36 ± 2.49 | 8.96 ± 1.96 | 2.39 ± 1.05 |
| Amphenicols | Florfenicol | 84.40 ± 2.4 | 85.18 ± 2.42 | 83.18 ± 2.52 | 83.18 ± 2.52 | 77.51 ± 2.89 |
| Sulfonamides | Sulphadimethoxine | 48.16 ± 3.39 | 49.07 ± 3.40 | 41.36 ± 3.32 | 26.41 ± 3.03 | 22.48 ± 2.89 |
| Folate pathway antagonist | Trimethoprim-sulfamethoxazole | 5.50 ± 1.54 | 8.33 ± 1.88 | 13.63 ± 2.31 | 10.37 ± 2.09 | 0.95 ± 0.67 |
