# Supplementary material for: Epidemiology of antimicrobial resistance (AMR) on California dairies: descriptive and cluster analyses of AMR phenotype of fecal commensal bacteria isolated from adult cows
Source: PeerJ. 2021 Apr 20;9:e11108. doi: 10.7717/peerj.11108 (PMC8063881; doi:10.7717/peerj.11108)
Supplement: Supplemental Information 9 [file peerj-09-11108-s009.docx]

Table S9. Proportion of resistance in *Escherichia coli* isolated from fecal samples of California dairy cows over sampling points during summer season.

| Antimicrobial class | Antimicrobial drug | Sampling points, days relative to calving | | | | |
| --- | --- | --- | --- | --- | --- | --- |
|  |  | Close-up | 30 | 60 | 90 | 120 |
| Penicillins | Ampicillin | 1.29 ± 0.74 | 1.31 ± 0.75 | 0.92 ± .065 | .098 ± 0.69 | 1.41± .081 |
| Cephalosporins | Ceftiofur | 1.29 ± 0.74 | 0.43 ± 0.43 | 0.00 ± 0.00 | 0.49 ± 0.49 | 0.94 ± 0.66 |
| Tetracyclines | Tetracycline | 2.06 ± 2.14 | 10.04 ± 1.99 | 8.75 ± 1.92 | 1.47 ± 2.48 | 15.09 ± 2.46 |
| Fluoroquinolones | Enrofloxacin | 3.87 ± 1.27 | 0.00 ± 0.00 | 0.00 ± 0.00 ^b^ | 0.00 ± 0.00 | 0.94 ± 0.66 |
|  | Danofloxacin | 4.31 ± 1.33 | 0.87 ± 0.61 | 0.46 ± 0.46 ^b^ | 0.49 ± 0.49 | 0.47 ± 0.47 |
| Aminoglycosides | Gentamicin | 0.00 ± 0.00 | 0.00 ± 0.00 | 0.00 ± 0.00 | 0.00 ± 0.00 | 0.00 ± 0.00 |
|  | Neomycin | 2.15 ± 0.95 | 0.87 ± 0.61 | 1.38 ± 0.79 | 1.47 ± 0.84 | 0.47 ± 0.47 |
|  | Spectinomycin | 3.01 ± 1.12 | 1.74 ± 0.86 | 1.38 ± 0.79 | 0.98 ± 0.69 | 1.41 ± 0.81 |
| Amphenicols | Florfenicol | 90.08 ± 1.96 | 82.96 ± 2.48 | 85.25 ± 2.41 | 82.84 ± 2.64 | 83.01 ± 2.58 |
| Sulfonamides | Sulphadimethoxine | 2.58 ± 1.04 | 0.43 ± 0.43 | 1.38 ± 0.79 | 0.98 ± 0.69 | 0.47 ± 0.47 |
| Folate pathway antagonist | Trimethoprim-sulfamethoxazole | 2.58 ± 1.04 | 0.43 ± 0.43 | 1.38 ± 0.79 | 0.98 ± 0.69 | 0.47 ± 0.47 |
