# Supplementary material for: Epidemiology of antimicrobial resistance (AMR) on California dairies: descriptive and cluster analyses of AMR phenotype of fecal commensal bacteria isolated from adult cows
Source: PeerJ. 2021 Apr 20;9:e11108. doi: 10.7717/peerj.11108 (PMC8063881; doi:10.7717/peerj.11108)
Supplement: Supplemental Information 10 — Due to difference in breakpoints for these drugs between Enterococcus spp. and Streptococcus spp. , the estimates should be interpreted with caution for Streptococcus spp. due to potential overestimation of the susceptibility. [file peerj-09-11108-s010.docx]

Table S10. Proportion of resistance in *Enterococcus* spp./ *Streptococcus* spp. isolates originated from fecal samples of California dairy cows over sampling points over two cohorts from 2018-2019.

| Antimicrobial class | Antimicrobial drug | Sampling point, days relative to calving | | | | |
| --- | --- | --- | --- | --- | --- | --- |
|  |  | Close-up | 30 | 60 | 90 | 120 |
| Penicillins | Ampicillin | 0.69 ± 0.40 | 0.22 ± 0.24 | 0.00 | 0.00 | 0.23 ± 0.24 |
|  | Penicillin | 0.23 ± 0.23 | 0.22 ± 0.22 | 0.00 | 0.00 | 0.47 ± 0.33 |
| Tetracyclines | Tetracycline | 14.15 ± 1.68 | 13.43 ± 1.62 | 17.23 ± 1.80 | 13.34 ± 1.64 | 18.13 ± 1.88 |
| Pleuromutilins | Tiamulin | 36.65 ± 2.32 | 43.05 ± 2.36 | 45.35 ± 2.37 | 42.15 ± 2.39 | 44.63 ± 2.43 |
| Macrolides | Gamithromycin | 11.83 ± 1.55 | 12.10 ± 1.55 | 15.64 ± 1.73 | 10.30 ± 1.47 | 7.64 ± 1.30 |
|  | Tilmicosin | 43.25 ± 2.40 | 46.24 ± 2.38 | 50.79 ± 2.38 | 48.711 ± 2.42 | 50.59 ± 2.44 |
|  | Tildipirosin | 45.01 ± 2.40 | 48.74 ± 2.38 | 53.10 ± 2.40 | 50.93 ± 2.42 | 53.22 ± 2.44 |
|  | Tulathromycin | 6.50 ± 1.18 | 7.74 ± 1.27 | 12.27 ± 1.56 | 7.25 ± 1.25 | 4.30 ± 0.99 |
|  | Tylosin | 1.15 ± 0.51 | 3.41 ± 0.86 | 6.57 ± 1.18 | 3.04 ± 0.83 | 1.67 ± 0.62 |
| Amphenicols | Florfenicol | 44.10 ± 2.39 | 46.01 ± 2.38 | 47.40 ± 2.38 | 47.54 ± 2.41 | 47.73 ± 2.44 |
