# Supplementary material for: Epidemiology of antimicrobial resistance (AMR) on California dairies: descriptive and cluster analyses of AMR phenotype of fecal commensal bacteria isolated from adult cows
Source: PeerJ. 2021 Apr 20;9:e11108. doi: 10.7717/peerj.11108 (PMC8063881; doi:10.7717/peerj.11108)
Supplement: Supplemental Information 11 — Due to difference in breakpoints for these drugs between Enterococcus spp. and Streptococcus spp., the estimates should be interpreted with caution for Streptococcus spp. due to potential overestimation of the susceptibility. [file peerj-09-11108-s011.docx]

Table S11. Proportion of resistance in *Enterococcus* spp./ *Streptococcus* spp. isolated from fecal samples of California dairy cows during different sampling points over winter cohort 2018-2019.

| Antimicrobial class | Antimicrobial drug | Sampling point, days relative to calving | | | | |
| --- | --- | --- | --- | --- | --- | --- |
|  |  | Close-up | 30 | 60 | 90 | 120 |
| Penicillins | Ampicillin | 1.53 ± 0.88 | 0.00 ± 0.00 | 0.00 ± 0.00 | 0.00 ± 0.00 | 0.47 ± 0.47 |
|  | Penicillin | 0.00 ± 0.00 | 0.47 ± 0.47 | 0.00 ± 0.00 | 0.00 ± 0.00 | 0.95 ± 0.67 |
| Tetracyclines | Tetracycline | 12.82 ± 2.40 | 11.37 ± 2.19 | 14.41 ± 2.36 | 11.21 ± 2.16 | 10.47 ± 2.11 |
| Pleuromutilins | Tiamulin | 30.76 ± 3.31 | 37.91 ± 3.34 | 38.73 ± 3.27 | 26.63 ± 3.02 | 39.04 ± 3.37 |
| Macrolides | Gamithromycin | 8.20 ± 1.97 | 9.95 ± 2.06 | 16.21 ± 2.47 | 10.74 ± 2.12 | 6.66 ± 1.72 |
|  | Tilmicosin | 28.86 ± 3.26 | 44.54 ± 3.42 | 42.79 ± 3.32 | 31.77 ± 3.19 | 40.00 ± 3.38 |
|  | Tildipirosin | 32.82 ± 3.37 | 46.44 ± 3.44 | 44.14 ± 3.40 | 34.11 ± 3.24 | 41.42 ± 3.40 |
|  | Tulathromycin | 4.61 ± 1.50 | 3.79 ± 1.30 | 10.81 ± 2.08 | 7.94 ± 1.85 | 3.80 ± 1.32 |
|  | Tylosin | 2.04 ± 1.01 | 2.36 ± 1.04 | 8.10 ± 1.83 | 4.20 ± 1.37 | 1.42 ± 0.82 |
| Amphenicols | Florfenicol | 29.74 ± 3.28 | 41.70 ± 3.40 | 42.34 ± 3.32 | 34.11 ± 3.24 | 46.19 ± 3.44 |
