# Supplementary material for: Epidemiology of antimicrobial resistance (AMR) on California dairies: descriptive and cluster analyses of AMR phenotype of fecal commensal bacteria isolated from adult cows
Source: PeerJ. 2021 Apr 20;9:e11108. doi: 10.7717/peerj.11108 (PMC8063881; doi:10.7717/peerj.11108)
Supplement: Supplemental Information 12 — Due to difference in breakpoints for these drugs between Enterococcus spp. and Streptococcus spp., the estimates should be interpreted with caution for Streptococcus spp. due to potential overestimation of the susceptibility. [file peerj-09-11108-s012.docx]

Table S12. Proportion of resistance in *Enterococcus* isolated from fecal samples of California dairy cows over sampling points during summer cohort.

| Antimicrobial class | Antimicrobial drug | Sampling points, days relative to calving | | | | |
| --- | --- | --- | --- | --- | --- | --- |
|  |  | Close-up | 30 | 60 | 90 | 120 |
| Penicillins | Ampicillin | 0.00 ± 0.00 | 0.43 ± 0.43 | 0.00 ± 0.00 | 0.00 ± 0.00 | 0.00 ± 0.00 |
|  | Penicillin | 0.42 ± 0.42 | 0.00 ± 0.00 | 0.00 ± 0.00 | 0.00 ± 0.00 | 0.00 ± 0.00 |
| Tetracyclines | Tetracycline | 15.25 ± 2.34 | 15.35 ± 2.39 | 20.09 ± 2.71 | 15.49 ± 2.48 | 25.83 ± 3.03 |
| Pleuromutilins | Tiamulin | 41.52 ± 3.21 | 47.80 ± 3.31 | 52.05 ± 3.38 | 57.74 ± 3.39 | 50.23 ± 3.46 |
| Macrolides | Gamithromycin | 14.83 ± 2.31 | 14.03 ± 2.30 | 15.06 ± 2.42 | 9.85 ± 2.04 | 8.61 ± 1.94 |
|  | Tilmicosin | 55.08 ± 3.24 | 47.80 ± 3.31 | 58.90 ± 3.33 | 65.72 ± 3.25 | 61.24 ± 3.37 |
|  | Tildipirosin | 55.08 ± 3.24 | 50.87 ± 3.31 | 62.10 ± 3.28 | 67.92 ± 3.21 | 65.07 ± 3.30 |
|  | Tulathromycin | 8.05 ± 1.77 | 11.40 ± 2.10 | 13.69 ± 2.32 | 6.57 ± 1.70 | 4.78 ± 1.47 |
|  | Tylosin | 0.42 ± 0.42 | 4.38 ± 1.35 | 5.02 ± 1.47 | 1.87 ± 0.93 | 1.91 ± 0.95 |
| Amphenicols | Florfenicol | 55.93 ± 3.23 | 50.00 ± 3.31 | 52.51 ± 3.38 | 61.03 ± 3.34 | 49.28 ± 3.46 |
